# *In situ* epoxide generation by dimethyldioxirane oxidation and the use of epichlorohydrin in the flow synthesis of a library of β-amino alcohols

**DOI:** 10.1098/rsos.171190

**Published:** 2018-04-04

**Authors:** Peter J. Cossar, Jennifer R. Baker, Nicholas Cain, Adam McCluskey

**Affiliations:** Chemistry, The University of Newcastle, University Drive Callaghan, New South Wales 2308, Australia

**Keywords:** dimethyl dioxirane, epoxide, flow chemistry, β-amino alcohols, microwave

## Abstract

The flow coupling of epichlorohydrin with substituted phenols, while efficient, limits the nature of the epoxide available for the development of focused libraries of β-amino alcohols. This limitation was encountered in the production of analogues of 1-(4-nitrophenoxy)-3-((2-((4-(trifluoromethyl)pyrimidin-2-yl)amino)ethyl)amino)propan-2-ol **1**, a potential antibiotic lead. The *in situ* (flow) generation of dimethyldoxirane (DMDO) and subsequent flow olefin epoxidation abrogates this limitation and afforded facile access to structurally diverse β-amino alcohols. Analogues of **1** were readily accessed either via (i) a flow/microwave hybrid approach, or (ii) a sequential flow approach. Key steps were the *in situ* generation of DMDO, with olefin epoxidation in typically good yields and a flow-mediated ring opening aminolysis to form an expanded library of β-amino alcohols **1** and **10a**–**18g**, resulting in modest (**11a**, 21%) to excellent (**12g**, 80%) yields. Alternatively flow coupling of epichlorohydrin with phenols **4a**–**4m** (22%–89%) and a Bi(OTf)_3_ catalysed microwave ring opening with amines afforded a select range of β-amino alcohols, but with lower levels of aminolysis regiocontrol than the sequential flow approach.

## 1. Introduction

**Figure 1.**
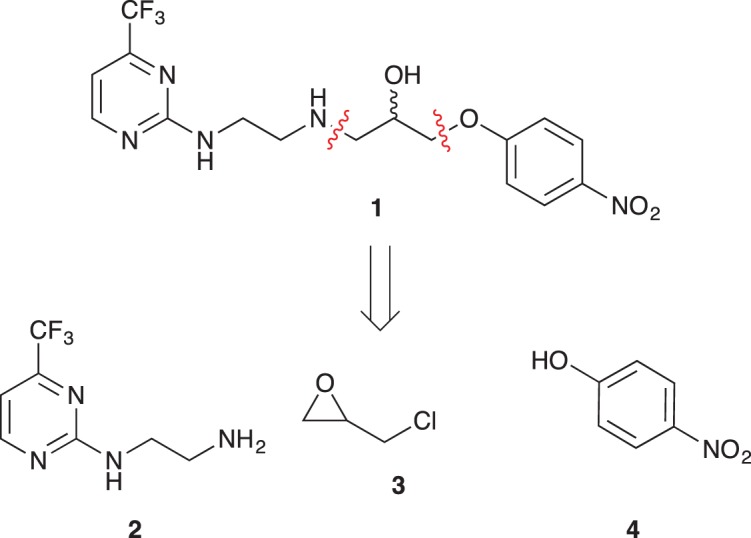
Chemical structure of lead **1** and the identification of three readily accessible fragments: aminopyrimidine **2**, epichlorohydrin **3** and 4-nitrophenol **4** for library development.

As part of our medicinal chemistry programme we identified β-amino alcohol **1** as a lead compound of interest. Analogues of this nature are known to be biologically active and have been used as key intermediates in the synthesis of natural products [1–5]. β-Amino alcohols are typically accessed through amine-mediated ring opening of epoxides, catalysed by Lewis acids [6–9]. In keeping with an epoxide ring opening strategy, **1** can be accessed via aminopyrimidine **2**, racemic epichlorohydrin **3** and 4-nitrophenol **4**, enabling robust focused library development (figure 1).

We like others have developed an interest in process intensification and streamlining of reaction optimization using flow chemistry approaches. Arguably, flow chemistry can now be considered a mature technology with considerable progress in the use of flow chemistry approaches in multi-step synthesis [10–20], the synthesis of drug like molecules [21–26], selective hydrogenations [27] and in the use of unstable and/or dangerous reagents [16–18,27,28].

To date, while the flow coupling of epichlorohydrin with phenols has been reported [29], the scope of this addition has been limited to a few examples only; as was the subsequent epoxide ring opening to form β-amino alcohols, a key constituent of active pharmaceutical ingredients (APIs) [23,30–36]. Critical to our proposed pathway was epoxide **3**, and for analogue development, related systems. Epoxides are known versatile intermediates giving rise to drugs such as pioglitazone, metoprolol and levofloxacin; or chemical probes such as wiskostatin [23,31,37,38]. Epoxide installation is typically by direct coupling of an epihalohydrin [37–40] or through *m*-chloroperoxybenzoic acid, hydrogen peroxide or *tert*-butyl hydroperoxide olefin epoxidation [41–43].

Herein we report a flow chemistry approach to epoxide installation using epichlorohydrin and the *in situ* epoxidation of olefins with dimethyldioxirane (DMDO) and the subsequent epoxide aminolysis, facilitating access to a focused library of β-amino alcohols based on **1** [44].

## 2. Results and discussion

**Scheme 1.**
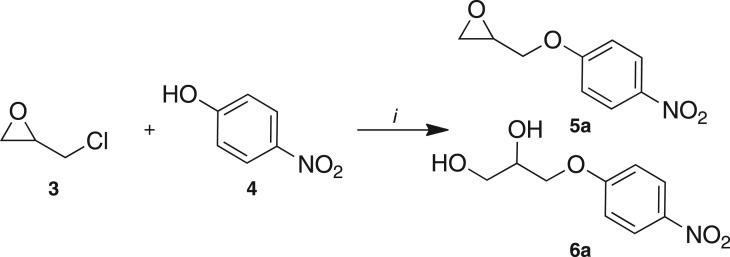
Reagents and conditions: (*i*) varying concentrations of **3** and 0.05 M **4** (DMF or CH_3_CN), base, 75–105°C, 1 ml min^−1^.

Method development commenced with the use of a flow instrument equipped with peristaltic pumps; passing two reagent streams, epichlorohydrin **3** (neat) and 4-nitrophenol **4** (0.1 M) in dimethylformamide (DMF), through an Omnifit® column packed with Cs_2_CO_3_ and acid washed sand (1 : 1) at 75°C, increasing in 10°C increments to 105°C, with the reaction monitored by UPLC-MS (scheme 1; table 1). The system pressure was maintained at 5 bar using the third pump in an active back pressure regulation mode, to circumvent the blockage issues caused by the precipitation of the product we had previously observed. This screen identified an enhanced coupling efficiency with **3** and **4** at higher temperatures, with DMF affording 97% at 105°C. Solvent switching to CH_3_CN/Cs_2_CO_3_ or DMF/diisopropylethyl amine (DIPEA) returned only unreacted **3** and **4**. DMF/1,8-diazabicyclo(5.4.0)undec-7-ene (DBU) and DMF/N(Bu)_4_OAc gave 90% and 100% conversion to **5a**, respectively. With DMF/N(Bu)_4_OAc, poor product recovery was a function of difficulties observed in isolating the desired product in the presence of N(Bu)_4_OAc; thus Cs_2_CO_3_ was used for on-going optimizations. Despite the ready recycling of epichlorohydrin, the use of reagents as solvents seriously limited future library generation and poses a greater environmental impact. Thus the effect of reducing the concentration of **3** on the conversion to **5a** was examined (table 2).

**Table 1.** Flow optimization of the coupling of epichlorohydrin **3** with 4-nitrophenol **4** at 0.5 ml min^−1^.

solvent	base	temp (°C)	ratio (**4** : **5a**)^a^^,^^b^
DMF	Cs_2_CO_3_	75	50 : 50
DMF	Cs_2_CO_3_	85	20 : 80
DMF	Cs_2_CO_3_	95	5 : 95
DMF	Cs_2_CO_3_	105	3 : 97
CH_3_CN	Cs_2_CO_3_	105	100 : 0
DMF	DIPEA	105	100 : 0
DMF	DBU	105	10 : 90
DMF	N(Bu)_4_OAc	105	0 : 100

^a^Reaction conditions: (*i*) 0.1 M 4-nitrophenol (**4**), neat epichlorohydrin (**3**), anhydrous DMF, residence time (*t*_r_) = 10 min, 5 bar.

^b^Ratios calculated by ultra performance liquid chromatography-tandem mass spectrometry (UPLC-MS) analysis.

**Table 2.** Flow optimization of the coupling of epichlorohydrin **3**, with 0.1 M 4-nitrophenol (**4**) at 1 ml min^−1^.

epichlorohydrin **3** (M)	residence time (min)	temp (°C)	ratio^a^^,^^b^ (**4** : **5a** : **6a**)
6	10	105	16 : 84 : 0
5	10	105	34 : 66 : 0
2.5	10	105	61 : 39 : 0
0.5	10	105	87 : 13 : 0
2.5	20	105	30 : 70 : 0
2.5	20	115	54 : 0 : 46
2.5	20	125	5 : 0 : 95
5	20	105	0 : 100 : 0

^a^ Reaction conditions: *t*_r_ = 10 min (10 ml loop) or 20 min (2 × 10 ml loop), 5 bar.

^b^Ratios calculated by UPLC-MS analysis.

**Scheme 2.**
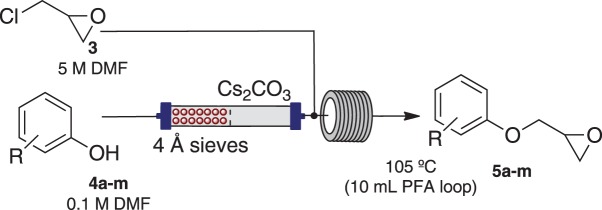
Reagents and conditions: anhydrous DMF, *t*_r_ = 20 min, 105°C, 5 bar, 1.0 ml min^−1^.

Use of 0.1 M of 4-nitrophenol **4** and **5**, 2.5 and 0.5 M **3** in DMF gave 66%, 39% and 13% conversion to **5a**, respectively. At 2.5 M of **3**, a 20 min residence time saw increased conversion to **5a** from 39% to 70%. At 115°C, unwanted diol **6a** was evident, but this was reduced through the use of a 4 Å sieves/acid washed sand/Cs_2_CO_3_ (2 : 1 : 1 v/v/v) loaded Omnifit® column. Quantitative conversion to epoxy ether **5a** was noted at 105°C, with 5 M **3** and a 20 min residence time (scheme 2).

With phenols **4a**–**4m** a range of conversion rates to the desired epoxy ether **5a**–**5m** from quantitative (**5a**, **5b** and **5d**) to modest (**5j**, 28%) were observed (table 3). Isolated yields in most cases were excellent (**5a**, **5d**, both 89%) with the exception of **5j** (22%, from 28% conversion). The remaining analogues showed consumption of phenol **4** but the presence of the undesired diol (**5c**, **5e** and **5m**), incomplete consumption of **4** (**5j**, **5k** and **5l**) or incomplete consumption of **4** and undesired diol (**5f**–**5i**) (table 3). In our hands the use of DMF and solid Cs_2_CO_3_ bypassed any potential issue with bi-phasic solutions, and the boiling point difference between DMF and epichlorohydrin facilitated easy removal and recycling of the latter reagent [29]. Racemic epichlorohydrin was used; while moderately inexpensive, the recycling procedure was considered necessary to reduce the environmental effects of excess epichlorohydrin, as well as demonstrate the use of the protocol if enantiomerically pure products were required.

**Table 3.** Flow synthesis of aromatic epoxy ethers **5a–5m**.

compound^a^	phenol (**4a**–**m**)	conversion (**4a**–**m **: **5a**–**m**: **6**)	yield (%)
**5a**	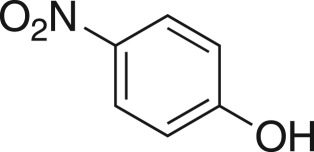	0 : 100 : 0	84–89 (*n* = 4)
**5b**	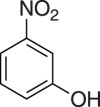	0 : 100 : 0	88
**5c**	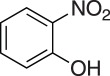	0 : 70 : 30	67
**5d**	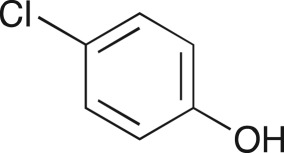	0 : 100 : 0	89
**5e**	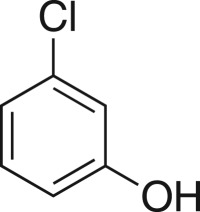	0 : 76 : 24	74
**5f**	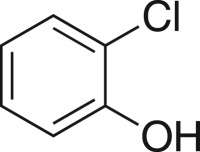	10 : 76 : 14	70
**5g**	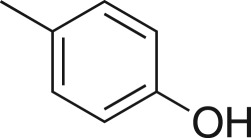	32 : 65 : 3	60
**5h**	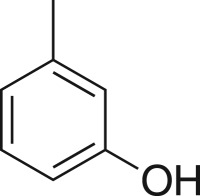	29 : 64 : 3	56
**5i**	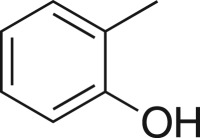	17 : 76 : 4	61
**5j**	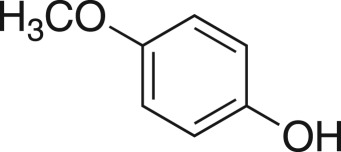	71 : 28 : 0	22
**5k**	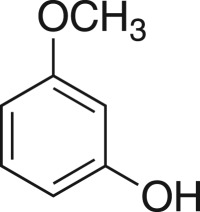	22 : 78 : 0	62
**5l**	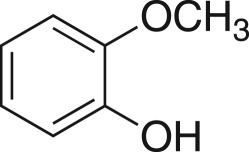	16 : 84 : 0	70
**5 m**	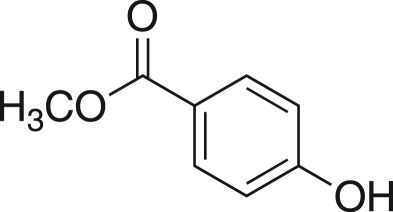	0 : 81 : 19	62

^a^For reagents and conditions, refer to scheme 2.

**Figure 2.**
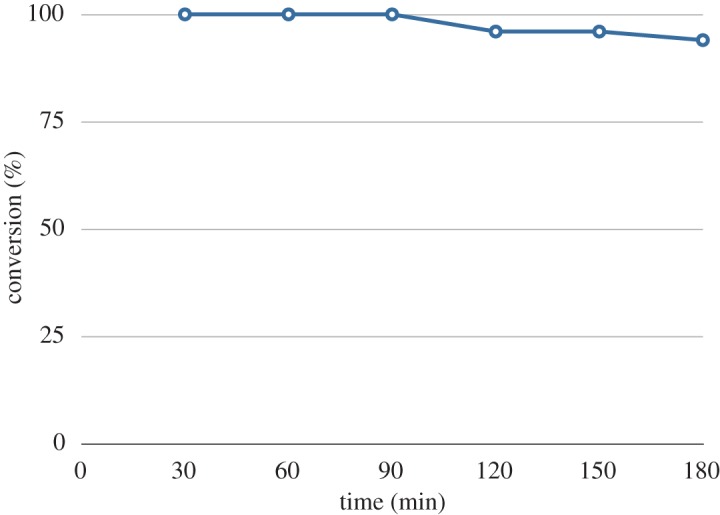
The percentage conversion of 5 M **3** and 0.1 M **4** (both in DMF) to **5a** at 1 ml min^−1^, 105°C as a function of time. Data collected at 30 min intervals, with conversions based on UPLC-MS analysis with ultraviolet-visible spectrometry detection at 320 and 255 nm.

Concerned about the possible depletion of the Cs_2_CO_3_ from the Omnifit® column, we scaled up the synthesis of **5a**, with a column packed with 16 g 1 : 1 w/w clean sand : Cs_2_CO_3_, examining the conversion rates as a function of time with analysis conducted at 30 min time points (figure 2). From this data, we were able to generate 1.48 g **5a** in 3 h (84% isolated yield; 0.49 g h^−1^). During the course of this continuous production the observed conversion rate remained essentially constant for the first 2.5 h, only showing a slight diminution after 3 h, to 94%. There was no evidence of channels of favoured flow paths developing within the Omnifit® column.

**Scheme 3.**
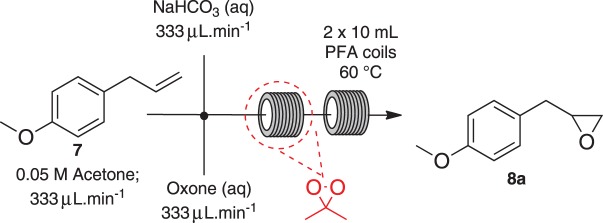
Reagents and conditions: 0.05 M **7** (acetone; 333 µl min^−1^), 0.315 M NaHCO_3_ (H_2_O, 333 µl min^−1^), Oxone® (H_2_O, 333 µl min^−1^), 60°C, 5 bar.

To nullify the limited availability of novel epoxides, we explored the *in situ* epoxidation capability of DMDO with selected aromatic and aliphatic olefins (table 4) [45,46]. The production of water-soluble by-products, as well as leveraging the known advantage of flow approaches in handling potentially sensitive reagents, made this method more attractive than traditional epoxidizing approaches [31,41–43]. No epoxidation of 4-allylanisole **7** with NaHCO_3_/Oxone® (1 : 1; *in situ* production of DMDO) was observed at 19°C, but at 30°C 16% conversion to epoxide **8a** was noted by gas chromatography mass spectrometry (GCMS) analysis (scheme 3). This increased to 61% at 60°C. Quantitative conversion to epoxide **8a** was accomplished using 2 equivalents of Oxone® at 60°C (electronic supplementary material, table S1). To further improve the safety of this reaction, the optimized reaction was repeated with the addition of a 0.4 M sodium sulfite_(aq)_ stream introduced after the back pressure regulator to quench any unreacted DMDO. Analysis and subsequent yield calculation showed no reduction in conversion or isolated yields.

**Table 4.** DMDO-mediated flow epoxidation of unsaturated scaffolds **8a**–**8k**.

alkene	product	conversion^a^ (**7** : **8**)	yield (%)
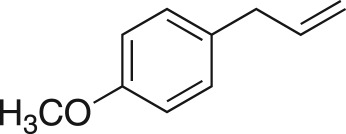	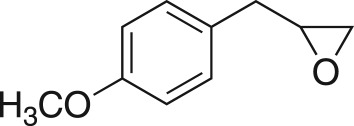	0 : 100	93
**7a**	**8a**		
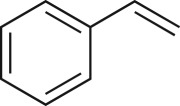	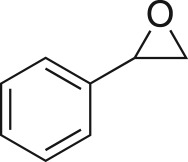	0 : 100	98
**7b**	**8b**		
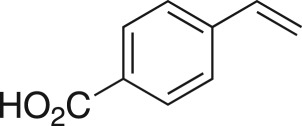	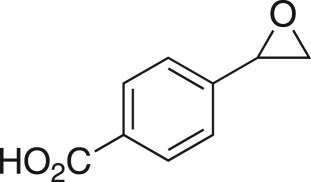	30 : 70	65
**7c**	**8c**		
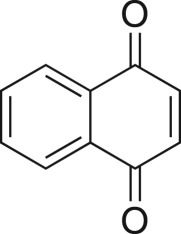	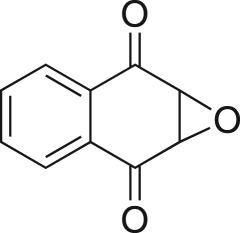	20 : 80	74
**7d**	**8d**		
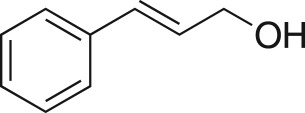	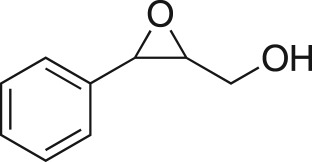	30 : 70	60
**7e**	**8e**		
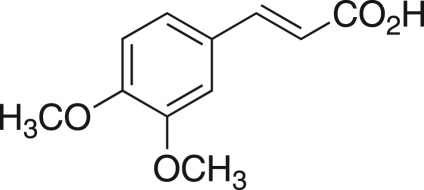	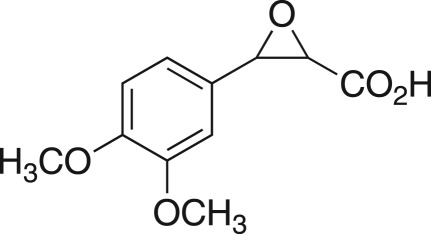	no reaction	—
**7f**	**8f**		
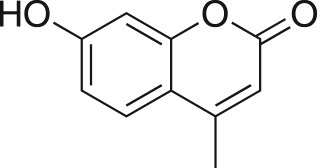	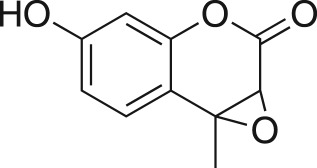	no reaction	—
**7g**	**8g**		
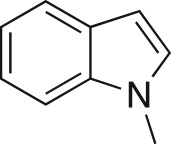	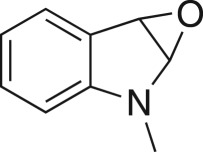	95 : 5	n.d.^b^
**7h**	**8h**		
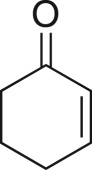	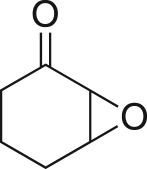	no reaction	—
**7i**	**8i**		
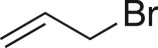	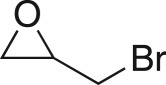	0 : 100	90
**7j**	**8j**		
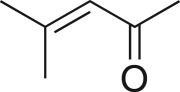	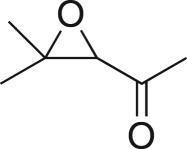	21 : 79	63
**7k**	**8k**		

^a^Conversion determine by ^1^H nuclear magnetic resonance spectroscopy (NMR).

^b^n.d., not determined.

The DMDO flow epoxidation protocol was explored with an auto-sampler and fraction collector equipped flow system and provided an effective and robust approach to the semi-automated synthesis and collection of epoxides via aromatic and aliphatic olefins (scheme 3 and table 4). Simple aromatic vinyl scaffolds showed good conversion (greater than 70%), with excellent isolated yields of benzyl epoxide **8a**, styrene oxides **8b** and **8c**, as did naphthoquinone and cinnamyl alcohol, yielding **8d** and **8e** (80% and 70%, respectively). By contrast, cinnamic acid **7f**, chromeone **7g**, indole **7h** and cyclohex-2-en-one **7i** scaffolds were resistant to DMDO epoxidation, with epoxides **8f**–**8i** at best observed at low levels (5% or less). Allyl bromide **7j** showed quantitative conversion to **8j**, while the highly substituted mesityl oxide **7k** showed a 79% conversion to mesityl epoxide **8 k** (table 4).

**Scheme 4.**
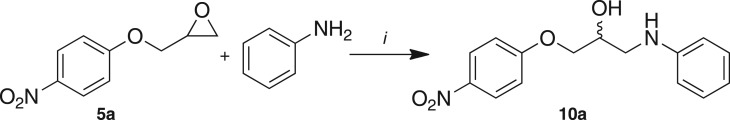
Reagents and conditions: (*i*) microwave irradiation, 1.1 equiv. aniline, 15% Bi(OTf)_3_, CH_3_CN, 10 min.

Having established two robust flow protocols to install epoxides via epichlorohydrin and DMDO, we next examined the amine-mediated ring opening of the freshly installed epoxide moiety. As we had previously reported similar amine mediated ring openings using bismuth (III) chloride as catalyst [8], we explored this approach; but to suppress the formation of chlorinated side products that can occur with this catalyst, bismuth (III) triflate (Bi(OTf)_3_) was examined. However, under flow conditions, employing 30 mol% Bi(OTf)_3_, 120°C, 5 bar and a residence time of 20 min, only a small amount (17%) of the amino alcohol was detected. Increasing the amount of bismuth catalyst to 50% increased the formation of product to 75%; however, as excess catalyst is undesirable for purification, we shifted focus to the application of microwave approaches (scheme 4) [44,47,48].

Coupling of epoxide **5a** with aniline and Bi(OTf)_3_ (15% in CH_3_CN) under microwave irradiation (60°C, 10 min) gave 57% conversion to the β-amino alcohol **10a** (electronic supplementary material, table S2). The optimal conditions were identified as 10 min microwave irradiation at 140°C with 15% Bi(OTf)_3_ in CH_3_CN, affording 96% conversion and an 83% isolated yield of **10a**. However, limited regiocontrol with other amines of interest was observed under these conditions (for ratio of regioisomers, see Experimental section).

**Scheme 5.**
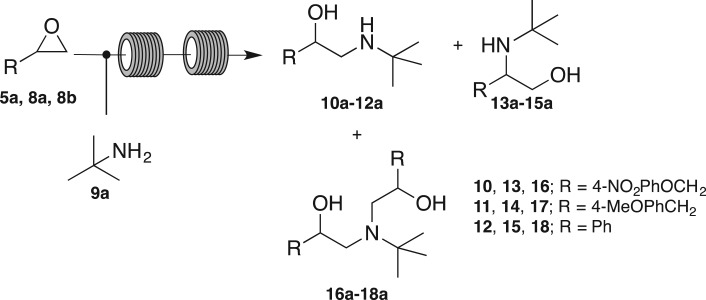
Reagents and conditions: epoxide **5a**, **8a** or **8b** (0.5 M in toluene) 0.5 ml min^−1^, *t*-butylamine (0.5 M in ethanol) 0.5 ml min^−1^, 120–150°C, 5–10 bar.

While the Bi-catalysed approach did allow access to the desired amino alcohols, the poor regioselectivity observed severely hampered compound access. We turned our attention to a recent report from the Seeberger laboratory that reported the model epoxide aminolysis of styrene with *t*-butyl and isopropyl amine by flow, [29] as well as a previous publication by Jamison and coworkers. [30]. While Seeberger's work reported the successful synthesis of a number of APIs under catalyst free conditions, the aminolysis step was limited to the aforementioned amines and the scope of the reaction was not defined. We endeavoured to extend the reach of these approaches. Application of the former protocols in our systems revealed that the optimum epoxide aminolysis conditions for aryl substituted epoxides ranged from 5–7 equivalents of *t*-butylamine, 120–150°C, 5–10 bar back pressure (scheme 5) and 1 : 1 *v*/*v* mixture of toluene : ethanol. These conditions afforded predominately the desired secondary amino alcohols (table 5).

**Table 5.** Optimized reaction conditions for flow aminolysis of epoxides **5a**, **8a** and **8b** with *t*-butylamine.

epoxide	*t*-butylamine (equiv.)	temp (°C)	pressure (bar)	conversion
**5a**	5	120	5	100 : 0 : 0
				**10a** **:** **13a** **:** **16a**
**8a**	5	150	10	80 : 0 : 20
				**11a** **:** **14a** **:** **17a**
**8b**	7	150	10	85 : 15: 0
				**12a** **:** **15a** **:** **18a**

Having established standard reaction conditions for aryl, aryl methyl and aryloxy epoxides, we applied this methodology across a range of primary, secondary and tertiary amines, as well as aniline, benzyl and *N*-benzylmethyl amines. Regioselective conversion to secondary alcohols **10b**–**10g** was observed with epoxide **5a** and amines **9b**–**9g** (table 6), as with the microwave protocol, and flow aminolysis yields ranged from modest to excellent (figure 2, 36%–80%). The exceptions to this were reactions of **5a** with **9a** and **9b**. Reaction of *N*-propylamine **9b** afforded a mixture of primary (**10b**), secondary alcohols (**13b**) and bis-alkylation (**16b**), while reaction of epoxide **5a** and *t*-butylamine **9a** furnished a 20% impurity of the bis-alkylated by-product (**16a**). Treatment of **5a** with *N*^1^-(4-(trifluoromethyl)pyrimidin-2-yl)ethane-1,2-diamine **2** regioselectively afforded exclusively **1** in a 65% isolated yield. This protocol delivered higher selectivity than microwave and batch methods, with no evidence of bis-alkylated side-product; [9] it has been reported that flow methodologies offer better selectivity over other methods owing to improved mixing [49].

**Table 6.** Flow aminolysis **5a**, **8a** and **8b** leading to the formation of amino alcohols **1** and (**10a**–**g**)–(**18a**–**g**).

						flow chemistry	microwave
epoxide	amine	2° alcohol (%)	1° alcohol (%)	bis-alkyl (%)	conversion (%)	isolated yield of 2° alcohol (%)	isolated yield of 2° alcohol (%)
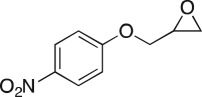	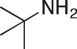	80	—	20	>99	66	—
**5a**	**9a**	**10a**	**13a**	**16a**		**10a**	
	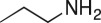	68	17	15	>99	not isolated	—
	**9b**	**10b**	**13b**	**16b**		—	
	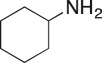	100	—	—	>99	75	—
	**9c**	**10c**	**13c**	**16c**		**10c**	
	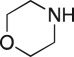	100	—	—	>99	36	40
	**9d**	**10d**	**13d**	**16d**		**10d**	
	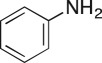	81	—	—	81	56	83
	**9e**	**10e**	**13e**	**16e**		**10e**	
	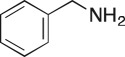	89	—	—	89	73	—
	**9f**	**10f**	**13f**	**16f**		**10f**	
	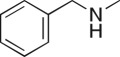	100	—	—	>99	80	53
	**9g**	**10g**	**13g**	**16g**		**10g**	
	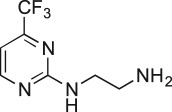	100	—	—	>99	63	45
	**2**	**1**	**13h**	**16h**		**1**	
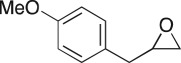		92	8	—	>99	21	—
**8a**	**9a**	**11a**	**14a**	**17a**		**11a**	
		—	>99			not isolated	—
	**9b**	**11b**	**14b**	**17b**		—	
		100	—	—	>99	49	—
	**9c**	**11c**	**14c**	**17c**		**11c**	
		100	—	—	>99	79	23
	**9d**	**11d**	**14d**	**17d**		**11d**	
		100	—	—	>99	59	IM^a^ 2 : 3
	**9e**	**11e**	**14e**	**17e**		**11e**	**11e** : **14e**^b^
		90	—	10	>99	50	—
	**9f**	**11f**	**14f**	**17f**		**11f**	
		100	—	—	>99	66	4 : 1*^a^*
	**9g**	**11g**	**14g**	**17g**		**11g**	**11g** : **14g***^b^*
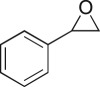		85	15	—	>99	49	—
**8b**	**9a**	**12a**	**15a**	**18a**		**12a**	
		11	65	24	>99	not isolated	—
	**9b**	**12b**	**15b**	**18b**		—	
		82	18	—	>99	58	—
	**9c**	**12c**	**15c**	**18c**		**12c**	
		100	—	—	>99	78	64
	**9d**	**12d**	**15d**	**18d**		**12d**	
		49	51	—	>99	35	32
	**9e**	**12e**	**15e**	**18e**		**12e**	
		85	12	3	>99	76	—
	**9f**	**12f**	**15f**	**18f**		**12f**	
		88	12	—	>99	60 (5 : 1*^a^* **12g** : **15g**)	
	**9g**	**12g**	**15g**	**18g**			

^a^IM, inseparable mixture.

^b^See the electronic supplementary material for experimental details.

Aminolysis of epoxide **8a** with amines **9a**–**9g** furnished **11a**–**11g** with ≥90% regioselectivity for the secondary alcohol, in principle simplifying purification of the desired regioisomer compared to the microwave methodology. However, the isolated yields of **11a**–**11g** ranged from 21% to 79%. LCMS analysis revealed product contamination with residual amine, and this was most apparent with aliphatic amines **9a** and **9c**.

Reaction of styrene epoxide **8b** with amines **9a**–**9g** showed reduced regioselectivity. The coupling of styrene epoxide **8b** with **9a** and **9f** afforded mixtures of primary (**12a** and **12f**) and secondary alcohols (**15a** and **15f**), as well as bis-alkylation (**18f**). Aniline **9e** afforded a 1 : 1 mixture of primary (**12e**) and secondary alcohols (**15e**), while the secondary amines **9b** and **9c**, as well as morpholine **9d**, benzyl amine **9f** and *N*-benzylmethylamine **9 g**, furnished the secondary alcohol in ≥82% regioselectivity. Yields of **12a**–**12f** ranged from modest to excellent (35–78%). As seen with epoxides **5a** and **8a**, reaction of *N*-propylamine **9b** resulted in a mixture of primary (**12b**), secondary (**15b**) and bis-alkyated products (**18b**). Compared with the microwave protocol, purification was improved because of improved regioselectivity (see Experimental section for regioisomer ratios). The notable exception to this proved to be **12g**, which was inseparable from **15g** by column chromatography, but offered comparable selectivity to the desired secondary alcohol as the microwave protocol.

## 3. Conclusion

Herein we have demonstrated that the flow coupling of phenols with epichlorohydrin provides a facile and highly scalable route to a wide variety of epoxides in moderate to excellent yields (22–89%). This access is scalable with multi-gram quantities of aryloxy epoxides accessible, e.g. **5a** (0.49 g h^−1^; with our three hour flow synthesis realizing 1.48 g of material). A microwave/Bi(OTf)_3_-mediated epoxide opening aminolysis gave moderate control over β-amino alcohol regiochemistry. However, our refinement of previously published aminolysis protocols [29,30] enabled development of a robust flow protocol with aryl, arylmethyl and aryloxy epoxides and a range of primary, secondary and tertiary amines, as well as aniline, benzyl and *N*-benzylmethyl amines. With this approach we were able to access a diverse range of β-amino alcohols with high regioselectivity. Further, we abrogated the limitations of this protocol, i.e. the use of epichlorohydrin, through the first application of flow DMDO olefin epoxidation, which was applicable to a wide range of aromatic and aliphatic olefins with isolated epoxide yields of 60–98%. The *in situ* generation of the reactive DMDO species improves the safety of the reaction compared with traditional batch methodologies, and semi-automation of this protocol using an auto-sampler and fraction collector facilitates rapid library generation. The in-flow epoxidation of olefins and flow epoxide aminolysis protocols developed herein offer the medicinal chemist a simple pathway to novel epoxides and, consequently, highly decorated β-amino alcohols of potential biological importance.

## 4. Experimental

### 4.1. General methods

All reagents were purchased from Sigma-Aldrich, AK Scientific, Matrix Scientific or Lancaster Synthesis and were used without purification. All solvents were used as supplied.

^1^H and ^13^C NMR spectra were recorded on a Brüker Advance™ AMX 400 MHz NMR spectrometer at 400.1 and 100.6 MHz, respectively or Brüker Advance™ AMX 600 MHz NMR spectrometer at 600.2 and 150.9 MHz, respectively. Chemical shifts (*δ*) are reported in parts per million (ppm) measured relative to the internal standards. Coupling constants (*J*) are expressed in hertz (Hz). Mass spectra were recorded on a Shimadzu LCMS 2010 EV or Agilent 6100 series single quadrupole LCMS using a mobile phase of 1 : 1 acetonitrile : H_2_O with 0.1% formic acid. The University of Wollongong, Australia, Mass Spectrometry User resource & Research Facility analysed samples for high resolution mass spectrometry (HRMS). All samples returned satisfactory analyses. Flow reactions were carried out using a Vapourtec RS-400 equipped with stainless steel pump module, fraction collection kit and auto-sampler; Vapourtec RS-200 equipped with V3 pumps, fraction collection kit and auto-sampler; and a Vapourtec easy-MedChem equipped with V3 pumps. Compound purity was confirmed by a combination of LCMS (UPLC), and HRMS and NMR analysis. All compounds are ≥95% purity.

### 4.2. Recycling procedure for epichlorohydrin

Epichlorohydrin was recovered and recycled by collecting post-reaction under vacuum (30 mbar, 50°C). The resulting crude mixture was then diluted with CH_2_Cl_2_ (100 ml). The solution was washed with water (3 × 150 ml) and saturated brine (150 ml). The organic layers were separated, dried over Mg_2_SO_4_ and concentrated *in vacuo* (40°C, 240 mbar) to afford a colourless oil.

### 4.3. General procedure 1

The Vapourtec easy-MedChem was charged with a 0.1 M solution of phenol (2 mmol) in anhydrous DMF (20 ml) at a flow rate of 0.5 ml min^−1^. The solution was then passed through a 7.85 ml Omnifit column packed with a 1 : 1 mixture of Cs_2_CO_3_/acid washed sand (2.30 g, total weight) and mixed with 5 M solution of epichlorohydrin in anhydrous DMF (0.5 ml min^−1^) through a T-piece. The resulting mixture was subsequently flowed through two 10 ml perfluroalkoxy (PFA) reaction coils set up in series at 105°C, 5 bar and affording a total of 20 min residence time. The resulting reaction mixture was collected, concentrated *in vacuo* and subject to column chromatography. The up-scaled reaction was carried out with the above concentration and an Omnifit column packed with a 1 : 1 mixture of Cs_2_CO_3_/acid (1 M HCl) washed sand (16 g, total mass).

### 4.4. 2-((4-Nitrophenoxy)methyl)oxirane (**5a**)

Compound **5a** was prepared using general procedure 1 and 4-nitrophenol (0.278 g, 2 mmol). The product was obtained as a light yellow solid (347 mg, 89%), m.p.: 66–69°C. ^1^H NMR (400 MHz, acetone-*d_6_*) *δ* 8.38–8.13 (m, 2H), 7.31–7.11 (m, 2H), 4.55 (dd, *J* = 11.4, 2.5 Hz, 1H), 4.05 (dd, *J* = 11.4, 6.5 Hz, 1H), 3.38 (ddt, *J* = 5.1, 4.4, 2.5 Hz, 1H), 2.91–2.84 (m, 1H), 2.76 (dd, *J* = 5.1, 2.5 Hz, 1H); ^13^C NMR (101 MHz, acetone-*d_6_*) *δ* 164.8, 142.6, 126.6 (2C), 115.8 (2C), 71.0, 50.2, 44.3; IR *υ*_max_/cm^−1^ 1504 (C–NO_2_), 1329 (C–NO_2_), 1250 (epox.) 1107 (C–O–C), 843 (arom.), 814 (epox.); low resolution mass spectrometry (LRMS) electron impact (EI) *m/z:* 195 (M^+^, 50%), 109 (35) 57 (100) [50].

### 4.5. 2-((3-Nitrophenoxy)methyl)oxirane (**5b**)

Compound **5b** was prepared using general procedure 1 and 3-nitrophenol (0.278 g, 2 mmol). The product was obtained as a light yellow solid (345 mg, 88%), m.p.: 55–58°C. ^1^H NMR (400 MHz, acetone-*d_6_*) *δ* 7.87 (ddd, *J* = 8.1, 2.1, 0.8 Hz, 1H), 7.81 (dd, *J* = 2.4, 2.1 Hz, 1H), 7.61 (t, *J* = 8.2 Hz, 1H), 7.45 (ddd, *J* = 8.3, 2.5, 0.7 Hz, 1H), 4.56 (dd, *J* = 11.4, 2.4 Hz, 1H), 4.05 (dd, *J* = 11.4, 6.5 Hz, 1H), 3.39 (ddt, *J* = 5.0, 4.3, 2.5 Hz, 1H), 2.89 (dd, *J* = 5.0, 4.3 Hz, 1H), 2.79 (dd, *J* = 5.1, 2.6 Hz, 1H); ^13^C NMR (101 MHz, acetone-*d_6_*) *δ* 160.3, 150.2, 131.4, 122.6, 116.6, 109.8, 71.0, 50.4, 44.3; IR *υ*_max_/cm^−1^: 1520 (C–NO_2_), 1346 (C–NO_2_), 1248 (epox.) 1034 (C–O–C), 813 (epox.), 797 (arom.); LRMS (EI) *m/z:* 195 (M^+^, 50%), 92 (35) 57 (100) [50].

### 4.6. 2-((2-Nitrophenoxy)methyl)oxirane (**5c**)

Compound **5c** was prepared using general procedure 1 and 2-nitrophenol (0.278 g, 2 mmol). The product was obtained as a yellow oil (125 mg, 67%). ^1^H NMR (400 MHz, acetone-*d_6_*) *δ* 7.84 (d, *J* = 8.1 Hz, 1H), 7.63 (dd, *J* = 11.5, 4.4 Hz, 1H), 7.37 (d, *J* = 8.5 Hz, 1H), 7.15 (t, *J* = 7.7 Hz, 1H), 4.55 (dd, *J* = 11.5, 2.2 Hz, 1H), 4.13 (dd, *J* = 11.4, 5.9 Hz, 1H), 3.42–3.27 (m, 1H), 2.87–2.83 (m, 1H), 2.78 (dd, *J* = 5.1, 2.6 Hz, 1H); ^13^C NMR (101 MHz, acetone-*d_6_*) *δ* 152.3, 141.3, 134.9, 125.8, 121.8, 116.2, 71.2, 50.2, 44.3; IR *υ*_max_/cm^−1^: 1521 (C–NO_2_), 1355 (C–NO_2_), 1259 (epox.) 1150 (C–O–C), 818 (epox.), 737 (arom.); LRMS (EI) *m/z:* 195 (M^+^, 10%), 57 (100) [50].

### 4.7. 2-((4-Chlorophenoxy)methyl)oxirane (**5d**)

Compound **5d** was prepared using general procedure 1 and 4-chlorophenol (0.257 g, 2 mmol). The product was obtained as a white solid (187 mg, 89%), m.p.: less than 40°C. ^1^H NMR (400 MHz, acetone-*d_6_*) *δ* 7.36–7.27 (m, 2H), 7.05–6.94 (m, 2H), 4.36 (dd, *J* = 11.3, 2.6 Hz, 1H), 3.89 (dd, *J* = 11.3, 6.4 Hz, 1H), 3.32 (ddt, *J* = 5.2, 4.2, 2.6 Hz, 1H), 2.85 (dt, *J* = 8.4, 4.2 Hz, 1H), 2.72 (dd, *J* = 5.1, 2.6 Hz, 1H); ^13^C NMR (101 MHz, acetone-*d_6_*) *δ* 158.6, 130.1 (2C), 126.1, 117.1 (2C), 70.5, 50.5, 44.3; IR *υ*_max_/cm^−1^: 1490 (arom.), 1239 (epox.) 1093 (C–O–C), 863 (arom.), 821 (epox.), 665 (C–Cl); LRMS (EI) *m/z:* 184 (M^+^, 80%), 128 (100) [51].

### 4.8. 2-((3-Chlorophenoxy)methyl)oxirane (**5e**)

Compound **5e** was prepared using general procedure 1 and 3-chlorophenol (0.257 g, 2 mmol). The product was obtained as a clear oil (228 mg, 74%). ^1^H NMR (400 MHz, acetone-*d_6_*) *δ* 7.29 (t, *J* = 8.1 Hz, 1H), 7.01 (t, *J* = 2.2 Hz, 1H), 6.98 (ddd, *J* = 8.1, 2.4, 0.7 Hz, 1H), 6.93 (dd, *J* = 8.4, 2.4 Hz, 1H), 4.37 (dd, *J* = 11.3, 2.5 Hz, 1H), 3.90 (dd, *J* = 11.3, 6.4 Hz, 1H), 3.31 (ddt, *J* = 5.1, 4.2, 2.6 Hz, 1H), 2.84 (dd, *J* = 4.9, 4.4 Hz, 1H), 2.72 (dd, *J* = 5.1, 2.6 Hz, 1H); ^13^C NMR (101 MHz, acetone-*d_6_*) *δ* 160.7, 135.3, 131.6, 121.8, 115.7, 114.4, 70.5, 50.4, 44.3; IR *υ*_max_/cm^−1^: 1475 (arom.), 1246 (epox.) 1072 (C–O–C), 858 (epox.), 767 (arom.), 679 (C–Cl); LRMS (EI) *m/z:* 184 (M^+^, 80%), 128 (100); HRMS electrospray ionisation (ESI) calcd for C_9_H_9_ClO_2_ (M^+^), 184.0291; no mass observed, material degraded.

### 4.9. 2-((2-Chlorophenoxy)methyl)oxirane (**5f**)

Compound **5f** was prepared using general procedure 1 and 2-chlorophenol (0.258 g, 2 mmol). The product was obtained as a clear oil (258 mg, 70%). ^1^H NMR (400 MHz, acetone-*d_6_*) *δ* 7.41 (dd, *J* = 7.9, 1.6 Hz, 1H), 7.30 (td, *J* = 7.9, 1.6 Hz, 1H), 7.16 (dd, 8.3, 0.9 Hz, 1H) 6.98 (td, *J* = 7.8, 1.4 Hz, 1H), 4.44 (dd, *J* = 11.4, 2.6 Hz, 1H), 4.02 (dd, *J* = 11.4, 6.1 Hz, 1H), 3.37 (ddt, *J* = 5.2, 4.2, 2.6 Hz, 1H), 2.87 (dd, *J* = 5.1, 4.3 Hz, 1H), 2.78 (dd, *J* = 5.2, 2.6 Hz, 1H); ^13^C NMR (101 MHz, acetone-*d_6_*) *δ* 155.2, 131.0, 129.0, 123.2, 122.7, 114.9, 70.9, 50.5, 44.3; IR *υ*_max_/cm^−1^: 1509 (arom.), 1238 (epox.) 1112 (C–O–C), 812 (epox.), 742 (arom.); LRMS (EI) *m/z:* 184 (M^+^, 40%), 128 (100) [41].

### 4.10. 2-((4-Tolyloxy)methyl)oxirane (**5g**)

Compound **5g** was prepared using general procedure 1 and *p*-cresol (210 µl, 2 mmol). The product was obtained as a clear oil (196 mg, 60%). ^1^H NMR (400 MHz, CDCl_3_) *δ* 7.09 (d, *J* = 8.4 Hz, 2H), 6.83 (d, *J* = 8.6 Hz, 2H), 4.18 (dd, *J* = 11.0, 3.3 Hz, 1H), 3.95 (dd, *J* = 11.0, 5.6 Hz, 1H), 3.37–3.32 (m, 1H), 2.98–2.81 (m, 1H), 2.75 (dd, *J* = 4.9, 2.7 Hz, 1H), 2.29 (s, 3H); ^13^C NMR (101 MHz, CDCl_3_) *δ* 156.5, 130.6, 130.1 (2C), 114.6 (2C), 69.0, 50.4, 44.9, 20.6; IR *υ*_max_/cm^−1^: 1600, 1582, 1491 (arom.), 1260 (epox.) 1154 (C–O–C), 857 (epox.), 770 (arom.); LRMS (EI) *m/z*: 164 (M^+^, 100%), 108 (90) [51].

### 4.11. 2-((3-Tolyloxy)methyl)oxirane (**5h**)

Compound **5h** was prepared using general procedure 1 and *m*-cresol (0.210 µl, 2 mmol). The product was obtained as a clear oil (184 mg, 56%). ^1^H NMR (400 MHz, CDCl_3_) *δ* 7.17 (t, *J* = 7.8 Hz, 1H), 6.79 (d, *J* = 7.5 Hz, 1H), 6.77–6.70 (m, 2H), 4.19 (dd, *J* = 11.0, 3.3 Hz, 1H), 3.96 (dd, *J* = 11.0, 5.6 Hz, 1H), 3.39–3.32 (m, 1H), 2.92–2.88 (m, 1H), 2.76 (dd, *J* = 4.9, 2.6 Hz, 1H), 2.33 (s, 3H); ^13^C NMR (101 MHz, acetone-*d_6_*) *δ* 159.8, 140.2, 130.1, 122.5, 116.1, 112.3, 70.0, 50.6, 44.4, 21.5; IR *υ*_max_/cm^−1^: 2882 (O–CH_3_), 1505 (arom.), 1221 (epox.) 1035 (C–O–C), 849 (arom.); LRMS (EI) *m/z*: 164 (M^+^, 100%), 108 (90) [50].

### 4.12. 2-((2-Tolyloxy)methyl)oxirane (**5i**)

Compound **5i** was prepared using general procedure 1 and *o*-cresol (0.210 µl, 2 mmol). The product was obtained as a clear oil (200 mg, 61%). ^1^H NMR (400 MHz, acetone-*d_6_*) *δ* 7.13 (bt, *J* = 6.7 Hz, 2H), 6.91 (d, *J* = 8.4 Hz, 1H), 6.84 (t, *J* = 7.4 Hz, 1H), 4.31 (dd, *J* = 11.3, 2.7 Hz, 1H), 3.91 (dd, *J* = 11.3, 6.0 Hz, 1H), 3.33 (ddd, *J* = 8.7, 4.2, 2.6 Hz, 1H), 2.83 (dd, *J* = 5.1, 4.3 Hz, 1H), 2.73 (dd, *J* = 5.2, 2.6 Hz, 1H), 2.20 (s, 3H); ^13^C NMR (101 MHz, acetone-*d_6_*) *δ* 157.8, 131.4, 127.8, 127.2, 121.5, 112.3, 70.0, 50.7, 44.4, 16.3; IR *υ*_max_/cm^−1^: 1497 (arom.), 1242 (epox.) 1121 (C–O–C), 837 (epox.), 749 (arom.); LRMS (EI) *m/z:* 164 (M^+^, 90%), 108 (100) [52].

### 4.13. 2-((4-Methoxyphenoxy)methyl)oxirane (**5j**)

Compound **5j** was prepared using general procedure 1 and 4-methoxyphenol (0.248 g, 2 mmol). The product was obtained as a white solid (79 mg, 22%), m.p.: 48 – 48°C. ^1^H NMR (400 MHz, CDCl_3_) *δ* 6.96–6.75 (m, 4H), 4.17 (dd, *J* = 11.0, 3.2 Hz, 1H), 3.92 (dd, *J* = 11.1, 5.6 Hz, 1H), 3.77 (s, 3H), 3.36–3.31 (m, 1H), 2.91–2.88 (m, 1H), 2.74 (dd, *J* = 4.9, 2.7 Hz, 1H); ^13^C NMR (101 MHz, dimethylsulfoxide (DMSO)-*d_6_*) *δ* 153.6, 152.3, 115.4 (2C), 114.6 (2C), 69.4, 55.3, 49.8, 43.7; IR *υ*_max_/cm^−1^: 2882 (O–CH_3_), 1505 (arom.), 1221 (epox.) 1035 (C–O–C), 849 (arom.), 823 (epox.); LRMS (EI) *m/z:* 180 (M^+^, 80%), 124 (100) [51].

### 4.14. 2-((3-Methoxyphenoxy)methyl)oxirane (**5k**)

Compound **5k** was prepared using general procedure 1 and 3-methoxyphenol (0.248 g, 2 mmol). The product was obtained as a clear oil (223 mg, 62%). ^1^H NMR (400 MHz, CDCl_3_) *δ* 7.18 (t, *J* = 8.1 Hz, 1H), 6.59–6.44 (m, 3H), 4.20 (dd, *J* = 11.0, 3.2 Hz, 1H), 3.95 (dd, *J* = 11.0, 5.6 Hz, 1H), 3.93 (s, 3H), 3.38–3.33 (m, 1H), 2.95–2.86 (m, 1H), 2.76 (dd, *J* = 4.9, 2.6 Hz, 1H); ^13^C NMR (101 MHz, DMSO-*d_6_*) *δ* 160.5, 159.5, 130.0, 106.6 (2C), 100.8, 69.0, 55.1, 49.7, 43.8; IR *υ*_max_/cm^−1^: 2833 (O–CH_3_), 1490 (arom.), 1198 (epox.) 1148 (C–O–C), 761 (arom.), 839 (epox.); LRMS (EI) *m/z*: 180 (M^+^, 90%), 124 (100) [52].

### 4.15. 2-((2-Methoxyphenoxy)methyl)oxirane (**5l**)

Compound **5l** was prepared using general procedure 1 and 2-methoxyphenol (0.248 g, 2 mmol). The product was obtained as a white solid (252 mg, 70%), m.p.: less than 40°C. ^1^H NMR (400 MHz, acetone-*d_6_*) *δ* 6.97 (dt, *J* = 8.0, 1.5 Hz, 2H), 6.92 (td, *J* *=* 7.7 Hz, 1H), 6.86 (td, *J* *=* 7.6, 1.8, 1H), 4.28 (dd, *J* = 11.3, 2.9 Hz, 1H), 3.88 (dd, *J* = 11.3, 6.2 Hz, 1H), 3.81 (s, 3H), 3.31 (ddd, *J* = 9.1, 4.2, 2.7 Hz, 1H), 2.82 (dd, *J* = 4.9, 4.1 Hz, 1H), 2.69 (dd, *J* = 5.2, 2.6 Hz, 1H); ^13^C NMR (101 MHz, DMSO-*d_6_*) *δ* 149.1, 147.7, 121.4, 120.7, 113.6, 112.2, 69.8, 55.4, 49.8, 43.8; IR *υ*_max_/cm^−1^: 2840 (O–CH_3_), 1508 (arom.), 1229 (epox.) 1023 (C–O–C), 860 (epox.), 745 (arom.); LRMS (EI) *m/z:* 180 (M^+^, 80%), 124 (100%) [42].

### 4.16. Methyl 4-(oxirane-2-ylmethoxy)benzoate (**5m**)

Compound **5m** was prepared using general procedure 1 and methyl 4-hydroxybenzoate (0.304 g, 2 mmol). The product was obtained as a white solid (228 mg, 67%), m.p.: 57–60°C. ^1^H NMR (400 MHz, acetone-*d_6_*) *δ* 8.06–7.85 (m, 2H), 7.17–6.84 (m, 2H), 4.45 (dd, *J* = 11.3, 2.6 Hz, 1H), 3.97 (dd, *J* = 11.3, 6.4 Hz, 1H), 3.84 (s, 1H), 3.34 (ddt, *J* = 6.7, 4.2, 2.6 Hz, 1H), 2.85 (dd, *J* = 5.0, 4.3 Hz, 2H), 2.74 (dd, *J* = 5.1, 2.6 Hz, 1H); ^13^C NMR (101 MHz, acetone-*d_6_*) *δ* 166.8, 163.5, 132.2 (2C), 123.8, 115.2 (2C), 70.4, 52.0, 50.4, 44.4; IR *υ*_max_/cm^−1^: 2882 (O–CH_3_), 1708 (C = O), 1508 (arom.), 1254 (epox.) 1025 (C–O–C), 847 (epox.); LRMS (EI) *m/z:* 208 (M^+^, 50%), 121 (100%) [53].

### 4.17. General procedure 2

Using a Vapourtec RS-200 equipped with collection valve kit and synthesis auto-sampler, a 10 ml reaction loop was charged with a 0.05 M solution of alkene (1 mmol) in acetone. The solution was pumped at 0.333 µl min^−1^ through PFA tubing and mixed with a stream of 0.315 M sodium hydrogen carbonate_(aq)_ (6.30 mmol) at 0.333 µl min^−1^. The outgoing solution then passed through an 8 cm PFA tube and mixed with a stream of 0.394 M Oxone_(aq)_ (7.88 mmol, 0.333 µl min^−1^). The resulting stream was subsequently passed through two 10 ml PFA coil reactors in series at 60°C, 5 bar and 1 ml min^−1^ (residence time 20 min). The resulting reaction stream was then quenched in-line (immediately after the back pressure regulator) using a stream 0.4 M sodium sulfite_(aq)_. The solution was then collected, concentrated *in vacuo* to remove acetone and diluted up to 50 ml with water (pH 7). The pH of the solution was adjusted to pH ∼7 using saturated ammonium chloride_(aq)_. The aqueous solution was extracted with ethyl acetate (3 × 50 ml), the organic layers were combined and washed with brine (50 ml). The organic layer was separated, dried over magnesium sulfate and concentrated *in vacuo* to afford the desired product; no further purification was required unless stated.

### 4.18. 2-(4-Methoxybenzyl)oxirane (**8a**)

Compound **8a** was prepared using general procedure 2 and 4-allylanisole (0.153 ml, 1 mmol). The product was obtained as a colourless oil (153 mg, 93%). ^1^H NMR (400 MHz, acetone-*d_6_*) *δ* 7.20 (d, *J* = 8.7 Hz, 2H), 6.87 (d, *J* = 8.7 Hz, 2H), 3.77 (s, 3H), 3.07–3.00 (m, 1H), 2.75 (dd, *J* = 5.5, 1.9 Hz, 2H), 2.68 (dd, *J* = 5.1, 4.0 Hz, 1H), 2.49 (dd, *J* = 5.2, 2.6 Hz, 1H); ^13^C NMR (101 MHz, acetone-*d_6_*) *δ* 159.5, 130.8 (2C), 130.5, 114.6 (2C), 55.5, 52.9, 46.7, 38.6; IR *υ*_max_/cm^−1^: 2836 (O–CH_3_), 1511 (arom.), 1243 (epox.) 1032 (C–O–C), 830 (arom.), 815 (epox.); LRMS (EI) *m/z:* 164 (M^+^, 30%), 121 (100%) [54].

### 4.19. 2-Phenyloxirane (**8b**)

Compound **8b** was prepared using general procedure 2 and styrene (0.114 ml, 1 mmol). The product was obtained as a clear oil (115 mg, 98%). ^1^H NMR (400 MHz, acetone-*d_6_*) *δ* 6.95–6.81 (m, 5H), 3.47 (dd, *J* = 4.0, 2.7 Hz, 1H), 2.66 (dd, *J* = 5.4, 4.2 Hz, 1H), 2.39 (dd, *J* = 5.4, 2.6 Hz, 1H); ^13^C NMR (101 MHz, acetone-*d_6_*) *δ* 128.0, 118.7 (2C), 118.4, 116.0 (2C), 41.8, 40.7; IR *υ*_max_/cm^−1^: 3046, 2990, 2910 (arom.), 873 (epox.) 755 (arom.); LRMS (EI) *m/z*: 120 (M^+^, 30%), 91 (100%) [55].

### 4.20. 4-(Oxiran-2-yl)benzoic acid (**8c**)

Compound **8c** was prepared using general procedure 2 and styrene (0.114 ml, 1 mmol). The product was obtained as an off-white solid (107 mg, 65%), m.p.: 132–133°C. ^1^H NMR (400 MHz, acetone-*d_6_*) *δ* 8.02 (d, *J* = 8.3 Hz, 2H), 7.44 (d, *J* = 8.3 Hz, 2H), 3.98 (dd, *J* = 3.9, 2.5 Hz 1H), 3.16 (dd, *J* = 5.6, 4.2 Hz, 1H), 2.81 (dd, *J* = 5.6, 2.5 Hz, 1H); ^13^C NMR (101 MHz, acetone-*d_6_*) *δ* 167.4, 144.4, 131.3, 130.6 (2C), 126.4 (2C), 52.1, 51.6; IR *υ*_max_/cm^−1^: 3057 (COOH) 2853 (arom.), 1681 (C=O), 1294 (C–O), 949 (C–O–C) 857 (epox.), 769 (arom.); LRMS (ESI^+^) *m/z*: 165 (M + H, 100%); HRMS (ESI) calcd for C_9_H_9_O_3_ (M + H), 165.0546; found 165.0547.

### 4.21. 1*α*,7α-dihydronaphtho[2,3-β]oxirene-2,7-dione (**8d**)

Compound **8d** was prepared using general procedure 2 and 1,4-naphthoquinone (0.158 g, 1 mmol). The product was obtained as a white solid (129 mg, 74%), m.p.: 132–134°C. ^1^H NMR (400 MHz, acetone-*d_6_*) *δ* 7.99 (dd, *J* = 5.7, 3.4 Hz, 2H), 7.89 (dd, *J* = 5.8, 3.3 Hz, 2H), 4.12 (s, 2H); ^13^C NMR (101 MHz, acetone-*d_6_*) *δ* 191.5 (2C), 135.5 (2C), 132.9 (2C), 127.6 (2C), 56.4 (2C); IR *υ*_max_/cm^−1^: 2878 (C–O), 1709 (C=O), 1683 (C=O), 1211 (epox.) 1027 (C–O–C), 880 (arom.), 818 (epox.); LRMS (EI) *m/z*: 174 (M^+^, 40%), 146 (40%) 105 (100%). [56]

### 4.22. (3-Phenyloxiran-2-yl)methanol (**8e**)

Compound **8e** was prepared using general procedure 2 and cinnamyl alcohol (0.130 ml, 1 mmol). The product was obtained as a clear oil (96 mg, 60%). ^1^H NMR (400 MHz, CDCl_3_) *δ* 7.39–7.27 (m, 5H), 4.05 (d, *J* = 12.7 Hz, 1H), 3.93 (d, *J* = 2.1 Hz, 1H), 3.85–3.77 (m, 1H), 3.26–3.21 (m, 1H), 1.89 (bs, 1H); ^13^C NMR (101 MHz, CDCl_3_) *δ* 136.8, 128.7 (2C), 128.5 (2C), 125.9, 62.5, 61.4, 55.7; IR *υ*_max_/cm^−1^: 3420 (OH), 1244 (epox.) 1025 (C–O–C), 750 (epox.); LRMS (EI) *m/z*: 150 (M^+^, 20%), 91 (100%) [55].

### 4.23. Epibromohydrin (8j)

Compound **8j** was prepared using general procedure 2 and allybromide (0.086 ml, 1 mmol). The product was obtained as a colourless oil (122 mg, 90%). ^1^H NMR (400 MHz, CDCl_3_) *δ* 3.43 (dd, *J* = 10.5, 5.9 Hz, 1H), 3.32 (dd, *J* = 10.5, 5.6 Hz, 1H), 3.29–3.24 (m, 1H), 2.96–2.92 (m, 1H), 2.67 (dd, *J* = 4.8, 2.4 Hz, 1H); ^13^C NMR (101 MHz, acetone-*d_6_*) *δ* 51.9, 48.8, 34.6; IR *υ*_max_/cm^−1^: 2921, 1464, (CH_2_), 1252, 1071 (epox.), 659 (C–Br); LRMS (EI) *m/z*: 120 (M^+^, 30%), 91 (100) [57].

### 4.24. 1-(3,3-Dimethyloxiran-2-yl)ethan-1-one (**8k**)

Compound **8k** was prepared using general procedure 2 and mesityl oxide (0.171 ml, 1.5 mmol). The product was obtained as a yellow oil (108 mg, 63%). ^1^H NMR (400 MHz, CD_3_OD) *δ* 3.60 (s, 1H), 2.23 (s, 3H), 1.42 (s, 3H), 1.22 (s, 3H); ^13^C NMR (101 MHz, acetone-*d_6_*) *δ* 204.0, 65.9, 60.8, 28.2, 24.7, 18.4; IR *υ*_max_/cm^−1^: 2971 (CH_3_), 1737 (C = O), 1217 (epox.) 1062 (C–O–C); LRMS (EI) *m/z*: 114 (M^+^, 5%), 99 (40), 43 (100) [58].

### 4.25. General procedure 3

A suspension of epoxide (1.00 mmol), amine (1.10 mmol) and bismuth (III) trifluoromethane sulfonate (15 mol%) in CH_3_CN (3 ml) was subjected to microwave irradiation at the below stated temperature and time (typical graph of the temperature/power/pressure in the electronic supplementary material, figure S1). The reaction mixture was concentrated *in vacuo* and crude material subjected to column chromatography.

### 4.26. General procedure 4

Using a Vapourtec RS-400 equipped with fraction collection kit and auto-sampler, either a 0.5 ml (loop A), 1.0 ml (loop B) or 2.0 ml (loop C) sample loop was charged with a 0.4 M solution of epoxide in toluene. An additional sample loop (either 0.5 ml (loop A), 1.0 ml (loop B) or 2.0 ml (loop C)) was charged with a 2.0 M amine solution in ethanol. The solutions were flowed together and the resulting stream was then passed through two 10 ml PFA coil reactors in series at 120°C, 6 bar back pressure and 1 ml min^−1^ (residence time 20 min). The resulting reaction mixture was collected and purified as described.

### 4.27. General procedure 5

Using a Vapourtec RS-400 equipped with fraction collection kit and auto-sampler, either a 0.5 ml (loop A), 1.0 ml (loop B) or 2.0 ml (loop C) sample loop was charged with a 0.4 M solution of epoxide in toluene. An additional sample loop (either 0.5 ml (loop A), 1.0 ml (loop B) or 2.0 ml (loop C)) was charged with either a 2.0 M or 2.8 M amine solution in ethanol (as stated). The solutions were flowed together and the resulting stream was then passed through two PFA coil reactors in series at 150°C, 10 bar back pressure and 1 ml min^−1^ (residence time 20 min). The resulting reaction mixture was collected and purified as described.

### 4.28. 1-(*tert*-Butylamino)-3-(4-nitrophenoxy)propan-2-ol (**10a**)

Compound **10a** was prepared using general procedure 4 with 2-((4-nitrophenoxy)methyl)oxirane (**5a**) (0.078 g, 0.4 mmol, loop B) and *tert*-butylamine (**9a**) (0.22 ml, 2.0 mmol, loop B). The resulting reaction mixture was concentrated using a stream of compressed air to remove solvent and volatile reagents to afford the desired product as a yellow solid (71 mg, 66%), m.p.: 80–83°C. ^1^H NMR (400 MHz, CD_3_OD) *δ* 8.27–8.17 (m, 2H), 7.17–7.09 (m, 2H), 4.18–4.01 (m, 3H), 2.84–2.70 (m, 2H), 1.16 (s, 9H); ^13^C NMR (151 MHz, CD_3_OD) *δ* 165.5, 143.0, 126.8 (2C), 115.9 (2C), 72.7, 70.0, 49.2, 46.0, 28.5 (3C); IR *υ*_max_/cm^−1^: 3302 (OH), 1648 (C–N), 1589 (C–NO_2_), 1329 (Ar–NO_2_), 843 (arom.); LRMS (ESI^+^) *m/z* 269 (100%, M + H); HRMS (ESI) calcd for C_13_H_21_N_2_O_4_ (M + H), 269.1496; found 269.1493.

### 4.29. 1-(Cyclohexylamino)-3-(4-nitrophenoxy)propan-2-ol (**10c**)

Compound **10c** was prepared using general procedure 4 with 2-((4-nitrophenoxy)methyl)oxirane (**5a**) (0.156 g, 0.8 mmol, loop C) and cyclohexylamine (**9c**) (0.46 ml, 4.0 mmol, loop C). The resulting reaction mixture was concentrated *in vacuo* and recrystallized from 1 : 1 *v/v* hexane : diethyl ether to afford the desired compound as a creamy white solid (168 mg, 71%), m.p.: 124–126°C. ^1^H NMR (400 MHz, CD_3_OD) *δ* 8.22 (d, *J* = 7.9 Hz, 2H), 7.12 (d, *J* = 7.9 Hz, 2H), 4.16–4.03 (m, 3H), 2.87 (d, *J* = 11.9 Hz, 1H), 2.77–2.66 (m, 1H), 2.53–2.41 (m, 1H), 1.94 (d, *J* = 11.5 Hz, 2H), 1.77 (d, *J* = 12.9 Hz, 2H), 1.66 (d, *J* = 11.5 Hz, 1H), 1.40–1.04 (m, 5H); ^13^C NMR (151 MHz, CD_3_OD) *δ* 165.5, 142.9, 126.8 (2C), 115.9 (2C), 72.8, 69.7, 58.1, 50.0, 33.94, 33.90, 27.2, 26.1; IR *υ*_max_/cm^−1^: 3247 (OH), 1593 (C–NO_2_), 1328 (Ar–NO_2_), 847 (arom.); LRMS (ESI^+^) *m/z* 295 (100%, M + H); HRMS (ESI) calcd for C_15_H_23_N_2_O_4_ (M + H), 295.1652; found 295.1649.

### 4.30. 1-Morpholino-3-(4-nitrophenoxy)propan-2-ol (**10d**)

Compound **10d** was prepared using general procedure 3; 2-((4-nitrophenoxy)methyl)oxirane (**5a**) (0.194 g, 1.00 mmol), morpholine (**9d**) (0.095 ml, 1.10 mmol) and bismuth (III) trifluoromethane sulfonate (0.100 g, 15 mol%) in CH_3_CN were subject to microwave irradiation at 140°C and 10 min. The resulting reaction mixture was concentrated *in vacuo*, adsorbed to silica and subjected to column chromatography (5% CH_3_OH in CH_2_Cl_2_) to afford the product as a cream solid (113 mg, 40%).

Compound **10d** was also prepared using general procedure 4 with 2-((4-nitrophenoxy)methyl)oxirane (**5a**) (0.039 g, 0.2 mmol, loop A) and morpholine (0.09 ml, 1.0 mmol, loop A). The resulting reaction mixture was concentrated *in vacuo* to afford the desired product as a brown solid (20 mg, 36%), m.p.: 80–88°C. ^1^H NMR (400 MHz, acetone-*d_6_*) *δ* 8.22 (d, *J* = 9.3 Hz, 2H), 7.17 (d, *J* = 9.3 Hz, 2H), 4.27 (q, *J* = 6.2 Hz, 1H), 4.20–4.04 (m, 3H), 3.62 (t, *J* = 4.7 Hz, 4H), 2.60–2.44 (m, 6H); ^13^C NMR (101 MHz, acetone-*d_6_*) *δ* 165.4, 142.3, 126.6 (2C), 115.8 (2C), 72.8, 67.5 (2C), 67.3, 62.1, 55.1 (2C); IR *υ*_max_/cm^−1^: 3302 (OH),, 1589 (C–NO_2_), 1329 (Ar–NO_2_), 843 (arom.); LRMS (ESI^+^) *m/z* 283 (100%, M + H) [59].

### 4.31. 1-(4-Nitrophenoxy)-3-(phenylamino)propan-2-ol (**10e**)

Compound **10e** was prepared using general procedure 3; 2-((4-nitrophenoxy)methyl)oxirane (**5a**) (0.194 g, 1.00 mmol), aniline (**9e**) (0.100 ml, 1.10 mmol) and bismuth (III) trifluoromethane sulfonate (0.100 g, 15 mol%) in CH_3_CN were subject to microwave irradiation at 140°C and 10 min. The resulting reaction mixture was concentrated *in vacuo*, adsorbed to silica and subjected to column chromatography (1.5% CH_3_OH in CH_2_Cl_2_) to afford the product as cream solid (238 mg, 82%).

Compound **10e** was also prepared using general procedure 4 with 2-((4-nitrophenoxy)methyl)oxirane (**5a**) (0.039 g, 0.2 mmol, loop A) and aniline (**9e**) (0.09 ml, 1.0 mmol, loop A). The resulting reaction mixture was concentrated using a stream of compressed air to remove solvent and volatile reagents to afford the desired product as a yellow solid (32 mg, 56%), m.p.: 126–130°C. ^1^H NMR (400 MHz, acetone-*d_6_*) *δ* 8.23 (d, *J* = 9.3 Hz, 2H), 7.18 (d, *J* = 9.3 Hz, 2H), 7.10 (dd, *J* = 8.3, 7.5 Hz, 2H), 6.70 (d, *J* = 8.4 Hz, 2H), 6.59 (t, *J* = 7.3 Hz, 1H), 5.02–4.91 (m, 1H), 4.49 (d, *J* = 4.3 Hz, 1H), 4.35–4.18 (m, 3H), 3.45 (ddd, *J* = 12.5, 6.4, 4.9 Hz, 1H), 3.34–3.23 (m, 1H); ^13^C NMR (101 MHz, acetone-*d_6_*) *δ* 165.2, 149.8, 142.4, 129.8 (2C), 126.6 (2C), 117.5, 115.8 (2C), 113.5 (2C), 72.3, 69.9, 47.21; IR *υ*_max_/cm^−1^: 3274 (OH), 1596 (C–NO_2_), 1330 (Ar–NO_2_), 843 (arom.); LRMS (ESI^+^) *m/z* 289 (100%, M + H) [60].

### 4.32. 1-(Benzylamino)-3-(4-nitrophenoxy)propan-2-ol (**10f**)

Compound **10f** was prepared using general procedure 4 with 2-((4-nitrophenoxy)methyl)oxirane (**5a**) (0.156 g, 0.8 mmol, loop C) and benzylamine (**9f**) (0.44 ml, 4.0 mmol, loop C). The resulting reaction mixture was concentrated *in vacuo*, adsorbed to silica and subjected to column chromatography (5% CH_3_OH in CH_2_Cl_2_) to afford the desired product as a yellow solid (138 mg, 57%), m.p.: 117–119°C. ^1^H NMR (400 MHz, DMSO-*d_6_*) *δ* 8.23–8.16 (m, 2H), 7.34–7.27 (m, 4H), 7.24–7.19 (m, 1H), 7.17–7.11 (m, 2H), 5.07 (d, *J* = 4.8 Hz, 1H), 4.16 (dd, *J* = 10.0, 4.1 Hz, 1H), 4.03 (dd, *J* = 10.0, 6.3 Hz, 1H), 3.97–3.90 (m, 1H), 3.76–3.68 (m, 2H), 2.61 (qd, *J* = 11.9, 5.9 Hz, 2H), 2.19 (s, 1H); ^13^C NMR (101 MHz, DMSO-*d_6_*) *δ* 164.2, 140.9, 140.7, 128.1 (2C), 127.9 (2C), 126.5, 125.9 (2C), 115.1 (2C), 71.7, 68.0, 53.1, 51.5; IR *υ*_max_/cm^−1^: 3284 (OH), 1594 (C–NO_2_), 1339 (Ar–NO_2_), 846 (arom.); LRMS (ESI^+^) *m/z* 303 (100%, M + H); HRMS (ESI) calcd for C_16_H_19_N_2_O_4_ (M + H), 303.1339; found 303.1336.

### 4.33. 1-(Benzyl(methyl)amino)-3-(4-nitrophenoxy)propan-2-ol (**10g**)

Compound **10g** was prepared using general procedure 3; 2-((4-nitrophenoxy)methyl)oxirane (**5a**) (0.194 g, 1.00 mmol), *N*-benzylmethylamine (**9 g**) (0.169 ml, 1.10 mmol) and bismuth (III) trifluoromethane sulfonate (0.100 g, 15 mol%) in CH_3_CN were subject to microwave irradiation at 140°C and 10 min. The resulting reaction mixture was concentrated *in vacuo*, adsorbed to silica and subjected to column chromatography (45% EtOAc in hexanes) to afford the product as a yellow oil (176 mg, 53%).

Compound **10g** was also prepared using general procedure 4 with 2-((4-nitrophenoxy)methyl)oxirane (**5a**) (0.039 g, 0.2 mmol, loop A) and *N*-benzylmethylamine (**9g**) (0.13 ml, 1.0 mmol, loop A). The resulting reaction mixture was concentrated *in vacuo* adsorbed to silica and subjected to column chromatography (45% EtOAc in hexanes) to afford the desired product as a brown oil (51 mg, 80%). ^1^H NMR (400 MHz, acetone-*d_6_*) *δ* 8.22 (d, *J* = 9.3 Hz, 2H), 7.42–7.19 (m, 5H), 7.13 (d, *J* = 9.3 Hz, 2H), 4.25 (dd, *J* = 9.4, 3.0 Hz, 1H), 4.21–4.04 (m, 3H), 3.60 (s, 2H), 2.61 (ddd, *J* = 27.6, 12.7, 6.3 Hz, 2H), 2.28 (s, 3H); ^13^C NMR (101 MHz, acetone-*d_6_*) *δ* 165.4, 142.3, 140.2, 129.8 (2C), 129.0 (2C), 127.8, 126.6 (2C), 115.8 (2C), 72.75, 68.0, 63.5, 60.6, 43.2; IR *υ*_max_/cm^−1^: 3431 (OH), 2842 (CH_2_), 1593 (C–NO_2_), 1511 (arom.), 1330 (Ar–NO_2_), 1112 (C–O–C), 1023 (C–O), 843 (arom.); LRMS (ESI^+^) *m/z* 331 (100%, M + H); HRMS (ESI) calcd for C_17_H_21_N_2_O_4_ (M + H), 317.1496; found 317.1494.

### 4.34. 1-(4-Nitrophenoxy)-3-[{2-(4-(trifluoromethyl)-2-pyrimidinyl)}amino]-2-propanol (**1**)

Compound **1** was prepared using general procedure 3; 2-((4-nitrophenoxy)methyl)oxirane (**5a**) (0.194 g, 1.00 mmol), **2** (0.101 ml, 1.10 mmol) and bismuth (III) trifluoromethane sulfonate (0.100 g, 15 mol%) in CH_3_CN were subject to microwave irradiation at 140°C and 5 min. The resulting reaction mixture was concentrated *in vacuo*, adsorbed to silica and subjected to column chromatography (1 : 10 : 89 NH_4_OH : CH_3_OH : CH_2_Cl_2_) to afford the desired product as a white solid (183 mg, 45%).

Compound **1** was also prepared using general procedure 5 with 2-((4-nitrophenoxy)methyl)oxirane (**5a**) (0.078 g, 0.4 mmol, loop B) and **2** (0.577 g, 2.8 mmol, loop B, 2.8 M), with a residence time of 40 min. The resulting reaction mixture was concentrated *in vacuo*, adsorbed to silica and subjected to column chromatography (1 : 10 : 89 NH_4_OH : CH_3_OH : CH_2_Cl_2_) to afford the desired product as a white solid (100 mg, 63%), m.p.: 130–131°C. ^1^H NMR (400 MHz, DMSO-*d_6_*) *δ* 8.59 (brs, 1H), 8.19 (d, *J* = 9.2 Hz, 2H), 7.78 (brd, *J* = 26.1 Hz, 1H), 7.14 (d, *J* = 9.3 Hz, 2H), 6.93 (d, *J* = 4.9 Hz, 1H), 5.06 (d, *J* = 3.9 Hz, 1H), 4.07 (ddd, *J* = 16.2, 10.0, 5.2 Hz, 2H), 3.89 (d, *J* = 3.9 Hz, 1H), 3.40–3.35 (m, 2H), 2.79–2.57 (m, 4H), 1.89 (brs, 1H); ^13^C NMR (151 MHz, CDCl_3_) *δ* 163.7, 162.7, 160.6, 156.6 (dd, *J* = 36.2, 36.8 Hz), 141.9, 126.1 (2C), 120.6 (dd, *J* = 550.4, 274.9 Hz), 114.7 (2C), 105.9 (d, *J* = 2.3 Hz), 71.2, 68.4, 51.4, 48.9, 41.4; IR *υ*_max_/cm^−1^: 3039 (OH), 1588 (C–NO_2_), 1515 (NH), 1341 (C–NO_2_), 1254 (C–F), 1130 (C–O–C), 848 (arom.); LRMS (ESI^+^) *m/z* 402 (M^+^, 100%); HRMS (ESI) calcd for C_16_H_18_F_3_N_5_O_4_ (M + H), 402.1384; found 402.1380.

### 4.35. 1-(tert-Butylamino)-3-(4-methoxyphenyl)propan-2-ol (**11a**)

Compound **11a** was prepared using general procedure 5 with compound **8a** (0.131 g, 0.8 mmol, loop C) and *tert*-butylamine (**9a**) (0.44 ml, 4.0 mmol, loop C, 2.0 M). The resulting reaction mixture was adsorbed to silica and subjected to column chromatography to afford the desired product as a brown solid (90 mg, 21%), m.p.: 44–46°C. ^1^H NMR (400 MHz, acetone-*d_6_*) *δ* 7.16 (d, *J* = 8.6 Hz, 2H), 6.86–6.79 (m, 2H), 3.75 (s, 3H), 3.69–3.61 (m, 1H), 2.69–2.61 (m, 3H), 2.40 (dd, *J* = 11.3, 7.9 Hz, 1H), 1.05 (s, 9H); ^13^C NMR (151 MHz, CDCl_3_) *δ* 178.2, 151.4, 150.3 (2C), 133.4 (2C), 91.6, 74.5, 69.5, 67.5, 60.8, 48.5 (3C); IR *υ*_max_/cm^−1^: 3297 (OH), 2928 (CH_2_, CH_3_), 1463 (arom.), 1244 (C–O), 1033 (C–O–C); LRMS (ESI^+^) *m/z* 238 (100%, M + H); HRMS (ESI) calcd for C_14_H_23_N_2_NaO_2_ (M + Na), 260.1626; found 260.1633.

### 4.36. 1-(Cyclohexylamino)-3-(4-methoxyphenyl)propan-2-ol (**11c**)

Compound **11c** was prepared using general procedure 5 with compound **8a** (0.131 g, 0.8 mmol, loop C) and cyclohexylamine (**9c**) (0.46 ml, 4.0 mmol, loop C, 2.0 M). The resulting reaction mixture was concentrated *in vacuo*, adsorbed to silica and subjected to column chromatography (10% CH_3_OH in CH_2_Cl_2_) to afford the product as a pale brown solid (53 mg, 40%), m.p.: 61–64°C. ^1^H NMR (400 MHz, CDCl_3_) *δ* 7.14 (d, *J* = 8.6 Hz, 2H), 6.84 (d, *J* = 8.6 Hz, 2H), 3.79–3.74 (m, 4H), 2.83–2.61 (m, 3H), 2.47 (dd, *J* = 12.0, 9.1 Hz, 1H), 2.39 (tt, *J* = 10.4, 3.7 Hz, 1H), 1.91–1.79 (m, 2H), 1.70 (d, *J* = 12.9 Hz, 2H), 1.65–1.54 (m, 1H), 1.28–1.11 (m, 3H), 1.09–0.97 (m, 2H); ^13^C NMR (101 MHz, CDCl_3_) *δ* 158.3, 130.5, 130.4 (2C), 114.0 (2C), 71.0, 56.8, 55.4, 51.8, 40.9, 34.0, 33.6, 26.2, 25.2, 25.1; IR *υ*_max_/cm^−1^: 3281 (OH), 2921 (CH_2_, CH_3_), 1461 (arom.), 1244 (C–O), 1038 (C–O–C); LRMS (ESI^+^) *m/z* 264 (100%, M + H); HRMS (ESI) calcd for C_16_H_25_NO_2_ (M + H), 264.1958; found 264.1956.

### 4.37. 1-(4-Methoxyphenyl)-3-morpholinopropan-2-ol (**11d**)

Compound **11d** was prepared using general procedure 3; compound **8a** (0.164 g, 1.0 mmol), morpholine (**9d**) (0.096 ml, 1.1 mmol) and bismuth (III) trifluoromethane sulfonate (0.100 g, 15 mol%) in CH_3_CN (2 ml) were subjected to microwave irradiation at 140°C for 10 min. The resulting reaction mixture was concentrated *in vacuo*, adsorbed to silica and subjected to column chromatography (50% EtOAc in hexanes) to afford the product as a pale brown oil (56 mg, 23%).

Compound **11d** was also prepared using general procedure 5 with compound **8a** (0.033 g, 0.2 mmol, loop A) and morpholine (**9d**) (0.09 ml, 1.0 mmol, loop A, 2.0 M). The resulting reaction mixture was concentrated *in vacuo* to afford the desired product as a brown oil (20 mg, 36%). ^1^H NMR (400 MHz, CDCl_3_) *δ* 7.14 (d, *J* = 8.6 Hz, 2H), 6.84 (d, *J* = 8.6 Hz, 2H), 3.93–3.88 (m, 1H), 3.79 (s, 3H), 3.70 (dt, *J* = 5.6, 3.8 Hz, 4H), 3.27 (bs, 1H), 2.77 (dd, *J* = 13.8, 6.9 Hz, 1H), 2.63 (dd, *J* = 13.6, 5.5 Hz, 3H), 2.41–2.30 (m, 4H); ^13^C NMR (101 MHz, CDCl_3_) *δ* 158.4, 130.4 (2C), 130.1, 114.0 (2C), 67.3, 66.9 (2C), 64.1 (2C), 55.4, 53.8, 40.5; IR *υ*_max_/cm^−1^: 3423 (OH), 2928, 2839 (CH_2_, CH_3_), 1511 (arom.), 1244 (C–O), 1115 (C–O–C); LRMS (ESI^+^) *m/z* 252 (100%, M + H); HRMS (ESI) calcd for C_14_H_22_NO_3_ (M + H), 252.1592; found 252.1595.

### 4.38. 1-(4-Methoxyphenyl)-3-(phenylamino)propan-2-ol 3 (**11e**)

A mixture **14e**/**11e** was prepared using general procedure 3; compound **8a** (0.164 g, 1.0 mmol), aniline (**9e**) (0.100 ml, 1.1 mmol) and bismuth (III) trifluoromethane sulfonate (0.100 g, 15 mol%) in CH_3_CN (2 ml) were subject to microwave irradiation at 140°C for 10 min. The resulting reaction mixture was concentrated *in vacuo*, adsorbed to silica and subjected to column chromatography (50% EtOAc in hexanes) to afford the product as a colourless oil (57 mg, 16%; ratio **14e** : **11e**, 3 : 2).

Compound **11e** was also prepared using general procedure 5 with compound **8a** (0.131 g, 0.8 mmol, loop C) and aniline (**9e**) (0.36 ml, 4.0 mmol, loop C, 2.0 M). The resulting reaction mixture was concentrated *in vacuo*, adsorbed to silica and subjected to column chromatography (5% CH_3_OH in CH_2_Cl_2_) to afford the desired product as a brown solid (121 mg, 59%), m.p.: 92–94°C. ^1^H NMR (400 MHz, CDCl_3_) *δ* 7.19–7.15 (m, 4H), 6.89–6.86 (m, 2H), 6.72 (t, *J* = 7.3 Hz, 1H), 6.63 (d, *J* = 7.6 Hz, 2H), 4.07–4.01 (m, 1H), 3.80 (d, *J* = 4.3 Hz, 3H), 3.31 (dd, *J* = 12.8, 3.4 Hz, 1H), 3.08 (dd, *J* = 12.8, 8.0 Hz, 1H), 2.84 (dd, *J* = 13.8, 5.2 Hz, 1H), 2.76 (dd, *J* = 13.8, 7.9 Hz, 1H); ^13^C NMR (101 MHz, CDCl_3_) *δ* 158.6, 148.4, 130.5 (2C), 129.7, 129.4 (2C), 118.0, 114.3 (2C), 113.4 (2C), 71.4, 55.4, 49.6, 40.8; IR *υ*_max_/cm^−1^: 3384 (OH), 2928 (CH_2_, CH_3_), 1493 (arom.), 1243 (C–O), 1031 (C–O–C); LRMS (ESI^+^) *m/z* 258 (100%, M + H); HRMS (ESI) calcd for C_16_H_20_NO_2_ (M + H), 258.1489; found 258.1489.

### 4.39. 1-(Benzylamino)-3-(4-methoxyphenyl)propan-2-ol (**11f**)

Compound **11f** was prepared using general procedure 5 with compound **8a** (0.131 g, 0.8 mmol, loop C) and benzylamine (**9c**) (0.46 ml, 4.0 mmol, loop C, 2.0 M). The resulting reaction mixture was concentrated *in vacuo*, adsorbed to silica and subjected to column chromatography (10% CH_3_OH in CH_2_Cl_2_) to afford the product as a pale brown solid (109 mg, 50%), m.p.: 63–66°C. ^1^H NMR (400 MHz, CD_3_OD) *δ* 7.33–7.22 (m, 5H), 7.11 (d, *J* = 8.6 Hz, 2H), 6.82 (d, *J* = 8.6 Hz, 2H), 3.93–3.87 (m, 1H), 3.79–3.68 (m, 5H), 2.72–2.61 (m, 3H), 2.56–2.50 (m, 1H); ^13^C NMR (101 MHz, CDCl_3_) *δ* 158.3, 140.1, 130.40 (2C), 130.36, 128.6 (2C), 128.2 (2C), 127.2 (2C), 114.0, 70.9, 55.4, 54.3, 53.8, 40.8; IR *υ*_max_/cm^−1^: 3200 (OH), 2936 (CH_2_), 2835 (O-CH_3_), 1510 (arom.), 1242 (C–O), 1033 (C–O–C); LRMS (ESI^+^) *m/z* 272 (100%, M + H); HRMS (ESI) calcd for C_17_H_22_NO_2_ (M + H), 272.1645; found 272.1642.

### 4.40. 1-(Benzyl(methyl)amino)-3-(4-methoxyphenyl)propan-2-ol (**11g**)

Compound **11g** was prepared using general procedure 3; compound **8a** (0.164 g, 1.0 mmol), *N*-benzylmethylamine (**9g**) (0.169 ml, 1.10 mmol) and bismuth (III) trifluoromethane sulfonate (0.100 g, 15 mol%) in CH_3_CN (2 ml) were subject to microwave irradiation at 140°C and 10 min. The resulting reaction was concentrated *in vacuo*, adsorbed to silica and subjected to column chromatography (10% CH_3_OH in CH_2_Cl_2_) to afford the product as a yellow oil (58 mg, 20%).

Compound **11g** was also prepared using general procedure 5 with compound **8a** (0.131 g, 0.8 mmol, loop C) and *N*-benzylmethylamine (**9g**) (0.103 ml, 4.0 mmol, loop C, 2.0 M). The resulting reaction mixture was adsorbed to silica and subjected to column chromatography (50% EtOAc in hexanes) to afford the desired product as a brown oil (150 mg, 66%). ^1^H NMR (400 MHz, acetone-*d_6_*) *δ* 7.39–7.22 (m, 5H), 7.17 (d, *J* = 8.7 Hz, 2H), 6.83 (d, *J* = 8.7 Hz, 2H), 3.97–3.88 (m, 1H), 3.77 (s, 3H), 3.63 (d, *J* = 13.2 Hz, 1H), 3.51 (d, *J* = 13.2 Hz, 1H), 2.67 (ddd, *J* = 20.9, 13.8, 6.1 Hz, 2H), 2.48–2.36 (m, 2H), 2.21 (s, 3H); ^13^C NMR (101 MHz, acetone-*d_6_*) *δ* 159.1, 140.0, 132.0, 131.2 (2C), 129.8 (2C), 129.0 (2C), 127.8, 114.3 (2C), 69.7, 64.0, 63.2, 55.4, 42.6, 41.4; IR *υ*_max_/cm^−1^: 3442 (OH), 2930 (CH_2_), 2837 (–O–CH_3_), 1512 (arom.), 1244 (C–O), 1032 (C–O–C); LRMS (ESI^+^) *m/z* 286 (100%, M + H); HRMS (ESI) calcd for C_18_H_24_NO_2_ (M + H), 286.1802; found 286.1800.

### 4.41. 2-(tert-Butylamino)-1-phenylethanol (**12a**)

Compound **12a** was prepared using general procedure 5 with styrene oxide (**8b**) (0.092 ml, 0.8 mmol, loop C) and *tert*-butylamine (**9a**) (0.59 ml, 5.6 mmol, loop C, 2.8 M). The resulting reaction mixture was adsorbed to silica and subjected to column chromatography to afford the desired product as a white solid (121 mg, 78%), m.p.: 85–87°C. ^1^H NMR (400 MHz, CD_3_OD) *δ* 7.41–7.31 (m, 4H), 7.30–7.23 (m, 1H), 4.70 (dd, *J* = 9.1, 4.0 Hz, 1H), 2.76 (dd, *J* = 11.4, 9.1 Hz, 1H), 2.69 (dd, *J* = 11.5, 4.0 Hz, 1H), 1.13 (s, 9H); ^13^C NMR (151 MHz, CD_3_OD) *δ* 165.5, 142.7, 126.8 (2C), 115.9 (2C), 72.7, 70.0, 46.0, 28.5 (3C); IR *υ*_max_/cm^−1^: 3288 (OH), 2973 (CH), 1605 (N–H), 1224 (C–O); LRMS (ESI^+^) *m/z* 194 (100%, M + H) [30].

### 4.42. 2-(Cyclohexylamino)-1-phenylethanol (**12c**)

Compound **12c** was prepared using general procedure 5 with styrene oxide (**8b**) (0.092, 0.8 mmol, loop C) and cyclohexylamine (**9c**) (0.46 ml, 4.0 mmol, loop C, 2.8 M). The resulting reaction mixture was concentrated *in vacuo*, adsorbed to silica and subjected to column chromatography (10% CH_3_OH in CH_2_Cl_2_) to afford the product as a crystalline tan solid (0.98 g, 58%), m.p.: 86–88°C. ^1^H NMR (400 MHz, acetone-*d_6_*) *δ* 7.39 (d, *J* = 7.2 Hz, 2H), 7.31 (t, *J* = 7.5 Hz, 2H), 7.23 (t, *J* = 7.2 Hz, 1H), 4.69 (dd, *J* = 9.1, 3.3 Hz, 1H), 2.93 (dd, *J* = 11.9, 3.6 Hz, 1H), 2.65 (dd, *J* = 11.9, 9.2 Hz, 1H), 2.52 (tt, *J* = 10.1, 3.7 Hz, 1H), 1.97–1.85 (m, 2H), 1.76–1.67 (m, 2H), 1.63–1.54 (m, 1H), 1.33–1.06 (m, 5H); ^13^C NMR (101 MHz, CDCl_3_) *δ* 144.9, 128.9 (2C), 127.8, 126.7 (2C), 72.6, 57.2, 55.6, 34.1, 33.8, 26.9, 25.6 (2C); IR *υ*_max_/cm^−1^: 3284 (OH), 2931 (CH), (N–H), 1492 (arom.), 1263 (C–O); LRMS (ESI^+^) *m/z* 220 (100%, M + H); HRMS (ESI) calcd for C_14_H_22_NO (M + H), 220.1696; found 220.1695.

### 4.43. 2-Morpholino-1-phenylethan-1-ol (**12d**)

Compound **12d** was prepared using general procedure 3; styrene oxide (**8b**) (0.114 ml, 1.0 mmol), morpholine (**9d**) (0.096 ml, 1.1 mmol) and bismuth (III) trifluoromethane sulfonate (0.100 g, 15 mol%) in CH_3_CN were subject to microwave irradiation at 140°C and 10 min. The resulting reaction was concentrated *in vacuo*, adsorbed to silica and subjected to column chromatography (5% CH_3_OH in CH_2_Cl_2_) to afford the product as a white solid (133 mg, 64%).

Compound **12d** was also prepared using general procedure 5 with styrene oxide (**8b**) (0.092 ml, 0.8 mmol, loop C) and morpholine (**9d**) (0.48 ml, 5.6 mmol, loop C, 2.8 M). The resulting reaction mixture concentrated *in vacuo* to afford the desired product as a brown solid (0.032 g, 78%), m.p.: 81–82°C. ^1^H NMR (400 MHz, acetone-*d_6_*) *δ* 7.40 (d, *J* = 7.6 Hz, 2H), 7.32 (t, *J* = 7.6 Hz, 2H), 7.23 (t, *J* = 7.2 Hz, 1H), 4.79 (dd, *J* = 9.0, 3.4 Hz, 1H), 4.10 (s, 1H), 3.72–3.58 (m, 4H), 2.69–2.60 (m, 2H), 2.52–2.41 (m, 4H); ^13^C NMR (101 MHz, acetone-*d_6_*) *δ* 144.5, 128.9 (2C), 128.0, 127.0 (2C), 69.9, 68.0 (2C), 67.5, 54.6 (2C); IR *υ*_max_/cm^−1^: 3122 (OH), 2926, 2817 (CH_2_, CH_3_), 1510 (arom.), 1134 (C–O), 1111 (C–O–C); LRMS (ESI^+^) *m/z* 208 (100%, M + H); HRMS (ESI) calcd for C_12_H_18_NO_2_ (M + H), 208.1332; found 208.1332.

### 4.44. 1-Phenyl-2-(phenylamino)ethan-1-ol (**12e**)

Compound **12e** was prepared using general procedure 3; styrene oxide (**8b**) (0.114 ml, 1.0 mmol), aniline (**9e**) (0.100 ml, 1.1 mmol) and bismuth (III) trifluoromethane sulfonate (0.100 g, 15 mol%) in CH_3_CN were subject to microwave irradiation at 140°C and 10 min. The resulting reaction mixture was concentrated *in vacuo*, adsorbed to silica and subjected to column chromatography (5% EtOAc in hexanes) to afford the product as a brown oil (68 mg, 32%).

Compound **12e** was also prepared using general procedure 5 with styrene oxide (**8b**) (0.092 ml, 0.8 mmol, loop C) and aniline (**9e**) (0.51 ml, 5.6 mmol, loop C, 2.8 M). The resulting reaction mixture was adsorbed to silica and subjected to column chromatography to afford the desired product as a brown oil (56 mg, 33%). ^1^H NMR (400 MHz, acetone-*d_6_*) *δ* 7.44 (d, *J* = 7.3 Hz, 2H), 7.30 (t, *J* = 7.5 Hz, 2H), 7.25–7.17 (m, 1H), 7.00 (dd, *J* = 8.5, 7.4 Hz, 2H), 6.61–6.49 (m, 3H), 5.33 (d, *J* = 3.0 Hz, 1H), 4.46 (dt, *J* = 8.0, 4.9 Hz, 1H), 4.06 (t, *J* = 5.9 Hz, 1H), 3.82 (ddd, *J* = 10.4, 5.7, 4.5 Hz, 1H), 3.66 (ddd, *J* = 11.0, 7.8, 6.0 Hz, 1H); ^13^C NMR (101 MHz, acetone-*d_6_*) *δ* 149.3, 142.8, 129.6 (2C), 129.2 (2C), 127.8 (3C), 117.5, 114.3 (2C), 67.8, 61.2; IR *υ*_max_/cm^−1^: 3391 (OH), 2928 (CH), 1601 (N–H), 1502 (arom.), 1263 (C–O); LRMS (ESI^+^) *m/z* 214 (100%, M + H) [30,61].

### 4.45. 2-(Benzylamino)-1-phenylethanol (**12f**)

Compound **12f** was prepared using general procedure 5 with styrene oxide (**8b**) (0.092 ml, 0.8 mmol, loop C) and benzylamine (**9f**) (0.61 ml, 5.6 mmol, loop C, 2.8 M). The resulting reaction mixture was adsorbed to silica and subjected to column chromatography to afford the desired product as a brown solid (138 mg, 76%), m.p.: 83–87°C. ^1^H NMR (400 MHz, CDCl_3_) *δ* 7.37–7.26 (m, 10H), 4.74 (dd, *J* = 8.9, 3.6 Hz, 1H), 3.89–3.81 (m, 2H), 2.95 (dd, *J* = 12.2, 3.6 Hz, 1H), 2.76 (dd, *J* = 12.2, 8.9 Hz, 1H); ^13^C NMR (101 MHz, CDCl_3_) *δ* 128.7, 128.5, 128.3, 127.7, 127.4, 126.0, 77.4, 71.9, 56.6, 53.6, 31.1; IR *υ*_max_/cm^−1^: 3289 (OH), 2933 (CH), 1626 (N–H), 1502 (arom.), 1299 (C–O).); LRMS (ESI^+^) *m/z* 228 (100%, M + H) [62].

### 4.46. 2-(Benzyl(methyl)amino)-2-phenylethanol (**12g**) :2-(benzyl(methyl)amino)-1-phenylethanol (**15g**)

A mixture **12g/15g** was prepared using general procedure 3; styrene oxide (**8b**) (0.114 ml, 1.00 mmol), *N*-benzylmethylamine (**9g**) (0.169 ml, 1.10 mmol) and bismuth (III) trifluoromethane sulfonate (0.100 g, 15 mol%) in 3 ml CH_3_CN were subject to microwave irradiation at 140°C and 10 min. The resulting reaction was concentrated *in vacuo*, adsorbed to silica and subjected to column chromatography (5% CH_3_OH in CH_2_Cl_2_) to afford the product as a yellow oil (0.126 g, 52%; ratio of **12 g** : **15 g**, 1 : 5).

Mixture **12g/15g** was also prepared using general procedure 5 with styrene oxide (**8b**) (0.092 ml, 0.8 mmol, loop C) and *N*-benzylmethylamine (**9g**) (0.72 ml, 5.6 mmol, loop C, 2.8 M). The resulting reaction mixture was adsorbed to silica and subjected to column chromatography to afford the desired product as a yellow oil (126 mg, 60%). Major regioisomer: ^1^H NMR (400 MHz, acetone-*d_6_*) *δ* 7.51–7.13 (m, 10H), 4.80 (dd, *J* = 8.9, 4.4 Hz, 1H), 4.11 (s, 1H), 3.72 (d, *J* *=* 13.4 Hz, 1H), 3.56 (d, *J* *=* 13.4 Hz, 1H), 2.64–2.47 (m, 2H), 2.30 (s, 3H); ^13^C NMR (101 MHz, acetone-*d_6_*) *δ* 144.6, 140.0, 129.8 (2C), 129.1 (2C), 128.8 (2C), 127.8, 127.8, 126.8 (2C), 70.7, 66.6, 63.0, 42.4; minor regioisomer: ^1^H NMR (400 MHz, acetone-*d_6_*) *δ* 7.57–7.08 (m, 10H), 4.07–4.02 (m, 1H), 3.79–3.73 (m, 2H), 3.62 (d, *J* *=* 13.4 Hz, 1H), 3.42 (d, *J* *=* 13.4 Hz, 1H), 2.13 (s, 3H); ^13^C NMR (101 MHz, acetone-*d_6_*) *δ* 129.8, 129.6, 128.9, 128.1, 127.7, 70.3, 62.9, 59.6, 38.2; IR *υ*_max_/cm^−1^: 3431 (OH), 2941 (CH_2_), 1442 (CH_2_), 1019 (C–O–C); LRMS (ESI^+^) *m/z* 242 (100%, M + H); HRMS (ESI) calcd for C_16_H_20_NO (M + H) 242.1539; found 242.1539.

## Supplementary Material

Compound synthesis and spectral characterisation

## Data accessibility

The datasets supporting this article have been uploaded as part of the electronic supplementary material.
