# Supplementary material for: In situ epoxide generation by dimethyldioxirane oxidation and the use of epichlorohydrin in the flow synthesis of a library of β-amino alcohols
Source: R Soc Open Sci. 2018 Apr 4;5(4):171190. doi: 10.1098/rsos.171190 (PMC5936892; doi:10.1098/rsos.171190)
Supplement: Compound synthesis and spectral characterisation [file rsos171190supp1.docx]

In situ epoxide generation by dimethyldioxirane oxidation and the use of epichlorohydrin in the flow synthesis of a library of β-amino alcohols

Peter J. Cossar,^†^ Jennifer R. Baker, ^†^ Nicholas Cain and Adam McCluskey*

Chemistry, School of Environmental & Life Sciences, The University of Newcastle, University Drive, Callaghan NSW 2308, Australia. Phone: +61(0)249216486; Fax: +61(0)249215472; Email: [Adam.McCluskey@newcastle.edu.au](mailto:Adam.McCluskey@newcastle.edu.au)

^†^These authors contributed equally to this work.

Table S1. The optimization of the epoxidation of 4-allylanisole 7 by dimethyldioxirane under flow chemistry conditions.

| Residence time (min) | Temp.  (°C) | Eq. Oxone | Eq. NaHCO_3_ | Ratio  (**7** : **8**) |
| --- | --- | --- | --- | --- |
| 20 | 19 (RT) | 1 | 3 | 100:0 |
| 20 | 30 | 1 | 3 | 84:16 |
| 20 | 40 | 1 | 3 | 54:46 |
| 20 | 50 | 1 | 3 | 44:56 |
| 20 | 60 | 1 | 3 | 39:61 |
| 20 | 60 | 1.5 | 5 | 20:80 |
| 20 | 60 | 2 | 6 | 0:100 |

Table S2. Optimization of the epoxide ring opening to form amino alcohol 10.

| Entry | Temp  (°C) | Catalyst | Conversion ^a^  SM : P | Isolated Yield (%) |
| --- | --- | --- | --- | --- |
| 1 | 60 | BiOTf | 43 : 57 |  |
| 2 | 80 | BiOTf | 42 : 58 |  |
| 3 | 100 | BiOTf | 32 : 68 |  |
| 4 | 120 | BiOTf | 19 : 81 |  |
| 5 | 140 | BiOTf | 4 : 96 | 83% |
| 6 | 140 | TfOH | -^b^ |  |
| 7 | 140 | -^c^ | 72 : 28 |  |

^a^ Conversion determined using UPLC-MS at 254 nm; ^b^ Polymerization; ^c^ no catalyst.


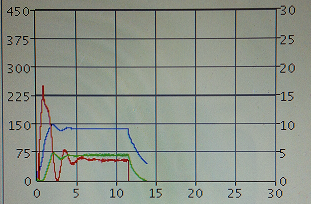


Figure S1. Representative graph of the temperature/power/pressure for the microwave-mediated Bi(OTf_3_)_3_-catalysed aminolysis. ^a^ Blue: Temperature (°C); Green: Pressure (bar); Red: Power (watt). Reaction time: 10 min.

**Experimental**

*3-(4-Methoxyphenyl)-2-(phenylamino)propan-1-ol* (**14e**) : *1-(4-methoxyphenyl)-3-(phenylamino)propan-2-ol* (**11e**) (3 : 2). A Mixture of **14e**/**11e** was prepared using general procedure 3; compound **8a** (0.164 g, 1.0 mmol), aniline (**9e**) (0.100 mL, 1.1 mmol) and bismuth (III) trifluoromethane sulfonate (0.100 g, 15 mol%) in CH_3_CN (~2 mL) were subject to microwave irradiation at 140 °C for 10 min. The resulting reaction mixture was concentrated *in vacuo*, adsorbed to silica and subjected to column chromatography (50% EtOAc in Hexane) to afford the product as a colourless oil (0.057 g, 16%). ^1^H NMR (400 MHz, CDCl_3_) δ 7.23 – 7.07 (m, 7.7H), 6.85 (dd, *J* = 12.3, 8.6 Hz, 3.9H), 6.79 – 6.62 (m, 5.8H), 4.09 – 4.00 (m, 0.8H), 3.80 (d, *J* = 4.5 Hz, 5.9H), 3.77 – 3.68 (m, 2.4H), 3.56 – 3.49 (m, 1.2H), 3.31 (dd, *J* = 12.8, 3.3 Hz, 0.9 H), 3.09 (dd, *J* = 12.8, 8.1 Hz, 1H), 2.93 – 2.72 (m, 4.6H); Major Regioisomer: ^13^C NMR (101 MHz, CDCl_3_) 158.5, 146.9, 130.4(2C), 129.7, 129.5(2C), 118.5, 114.3 (2C), 113.8(2C), 63.2, 56.6, 55.4, 36.4. Minor Regioisomer; ^13^C NMR (101 MHz, CDCl_3_) δ 158.6, 147.9, 130.5 (2C), 129.8, 129.6 (2C), 118.7, 114.5 (2C), 114.2 (2C), 71.3, 55.4, 49.9, 40.8 (inseparable mixture of regioisomers 3 : 2 (**14e** : **11e**); LRMS (ESI^+^) *m/z* 258 (100%, M+H).

*2-(Benzyl(methyl)amino)-2-phenylethanol* **(12g) :** *2-(Benzyl(methyl)amino)-1-phenylethanol* **(15g)**

A mixture of **12g/15g** was prepared using general procedure 3; styrene oxide (**8b**) (0.114 mL, 1.00 mmol), *N*-methyl benzylamine (**9g**) (0.169 mL, 1.10 mmol) and bismuth (III) trifluoromethane sulfonate (0.100 g, 15 mol%) in CH_3_CN were subject to microwave irradiation at 140 °C and 10 min. The resulting reaction was concentrated *in vacuo*, adsorbed to silica and subjected to column chromatography (5% MeOH in DCM) to afford the product as a yellow oil (0.126 g, 52%). Major regioisomer: ^1^H NMR (400 MHz, acetone-*d_6_*) δ 7.51 – 7.13 (m, 10H), 4.80 (dd, *J* = 8.9, 4.4 Hz, 1H), 4.11 (s, 1H), 3.72 (d, *J =* 13.4 Hz, 1H), 3.56 (d, *J =* 13.4 Hz, 1H), 2.64 – 2.47 (m, 2H), 2.30 (s, 3H); ^13^C NMR (101 MHz, acetone-*d_6_*) δ 144.6, 140.0, 129.8 (2C), 129.1 (2C), 128.8 (2C), 127.8, 127.8, 126.8 (2C), 70.7, 66.6, 63.0, 42.4; Minor regioisomer: ^1^H NMR (400 MHz, acetone-*d_6_*) δ 7.57 – 7.08 (m, 10H), 4.07 – 4.02 (m, 1H), 3.79 – 3.73 (m, 2H), 3.62 (d, *J =* 13.4 Hz, 1H), 3.42 (d, *J =* 13.4 Hz, 1H), 2.13 (s, 3H); ^13^C NMR (101 MHz, acetone-*d_6_*) δ 129.8, 129.6, 128.9, 128.1, 127.7, 70.3, 62.9, 59.6, 38.2.

H_2_O

**1**

**1**

**15g**

**12g**

**12g**

**15g**
